# Multicenter integration analysis of TRP channels revealed potential mechanisms of immunosuppressive microenvironment activation and identified a machine learning‐derived signature for improving outcomes in gliomas

**DOI:** 10.1111/cns.14816

**Published:** 2024-07-01

**Authors:** Yuntao Li, Yonggang Zhang, Shi Feng, Hua Zhu, Ying Xing, Xiaoxing Xiong, Qianxue Chen

**Affiliations:** ^1^ Department of Neurosurgery Renmin Hospital of Wuhan University Wuhan China; ^2^ Central Laboratory Renmin Hospital of Wuhan University Wuhan China; ^3^ Department of Gastroenterology, 72nd Group Army Hospital Huzhou University Huzhou Zhejiang China

**Keywords:** gliomas, machine learning, TRP channel, tumor immune microenvironment

## Abstract

**Aim:**

This study aimed to explore the mechanisms of transient receptor potential (TRP) channels on the immune microenvironment and develop a TRP‐related signature for predicting prognosis, immunotherapy response, and drug sensitivity in gliomas.

**Methods:**

Based on the unsupervised clustering algorithm, we identified novel TRP channel clusters and investigated their biological function, immune microenvironment, and genomic heterogeneity. In vitro and in vivo experiments revealed the association between TRPV2 and macrophages. Subsequently, based on 96 machine learning algorithms and six independent glioma cohorts, we constructed a machine learning‐based TRP channel signature (MLTS). The performance of the MLTS in predicting prognosis, immunotherapy response, and drug sensitivity was evaluated.

**Results:**

Patients with high expression levels of TRP channel genes had worse prognoses, higher tumor mutation burden, and more activated immunosuppressive microenvironment. Meanwhile, TRPV2 was identified as the most essential regulator in TRP channels. TRPV2 activation could promote macrophages migration toward malignant cells and alleviate glioma prognosis. Furthermore, MLTS could work independently of common clinical features and present stable and superior prediction performance.

**Conclusion:**

This study investigated the comprehensive effect of TRP channel genes in gliomas and provided a promising tool for designing effective, precise treatment strategies.

## Graphical abstract

To identify novel TRP channel gene clusters and describe their characteristics, we conducted an unsupervised clustering algorithm. Based on eight independent glioma cohorts and ten machine learning algorithms, a TRP‐related signature was constructed named MLTS. It provides a well‐developed and robust model to predict prognosis and immunotherapy response in gliomas.

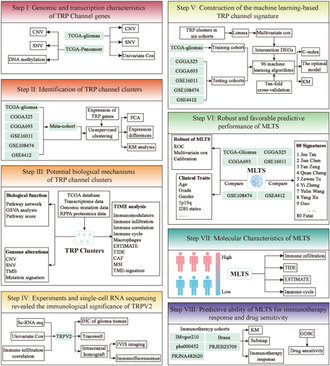

## 1 INTRODUCTION

Glioma is the most common primary intracranial tumor.^1^ The World Health Organization (WHO) classifies gliomas into four pathological grades (WHO I–IV). WHO II and III are low‐grade gliomas (LGG), and WHO IV is glioblastoma (GBM).^2^ Patients with LGG have an overall survival (OS) of 8–10 years. GBM is the most common primary malignant tumor of the central nervous system. The OS of patients with GBM is only 12–14 months.^3^ Despite progress in the clinical treatment of GBM, involving advances in surgery combined with radiotherapy and chemotherapy, the patient's 5‐year survival rate remains low due to the high invasiveness of the tumor and chemotherapy resistance.^4^, ^5^, ^6^ Therefore, effective solutions for the treatment of gliomas are urgently needed. Gliomas exhibit immunosuppressive properties that modulate antitumor immune responses.^3^, ^7^ Previous studies have shown that the therapeutic inhibition of indoleamine 2,3‐dioxygenase 1, cytotoxic T lymphocyte antigen 4, and PD ligand 1 in glioma models significantly reduced the tumor‐infiltrating Treg cells and improved long‐term survival, indicating immune checkpoint blockade appears to be a promising strategy for glioma immunotherapy.^3^, ^8^ However, the results of immune checkpoint blockade in clinical trials were not satisfactory.^9^ Therefore, taking the glioma TIME into consideration to identify new treatment targets and form a new prognosis assessment system has the potential to improve the prediction of patient prognosis.

The mammalian transient receptor potential (TRP) superfamily consists of six subfamilies and is mainly permeable to Ca^2+^ and monovalent cations.^10^, ^11^, ^12^ As a second messenger, Ca^2+^ participates in various cellular processes, including cell proliferation, migration, and apoptosis.^12^, ^13^ The imbalance in Ca^2+^ homeostasis is an intrinsic factor in various diseases. TRP channels have been implicated in a variety of central nervous system diseases, including glioma.^14^, ^15^, ^16^, ^17^ TRPC1 gene was shown to affect the proliferation and migration of glioma cells by regulating calcium transport.^18^, ^19^ In addition, TRPC6 was reported to be a crucial mediator of Notch‐driven glioblastoma growth and invasiveness.^20^ TRP channels are also involved in immunity. TRPV2 is related to macrophage function, and the loss of TRPV2 affects macrophage particle binding and phagocytosis.^21^ TRPML1 also contributes to phagosome–lysosome fusion and mediates phagocytosis of pathogens by macrophages.^22^ TRPV1 regulates pro‐inflammatory cytokines in bronchial epithelial cells, and the loss of TRPV1 inhibits LPS‐induced activation of peritoneal macrophages.^23^ Hence, the TRP channel genes (TRPGs) have indeed shown great potential for immunotherapy applications. However, the role of these genes in glioma immunity remains unclear.

In this study, we collected 20 TRPGs and identified two TRP channel clusters using unsupervised clustering to explore their genomic and transcriptomic heterogeneity in gliomas. Moreover, we conducted in vitro and in vivo experiments to demonstrate that TRPV2 activation could promote macrophage migration toward malignant cells, and improve glioma prognosis. Finally, we performed a prognostic machine learning‐based signature of the TRP channels in gliomas, which had a robust and favorable predictive ability for prognosis, immune infiltration status, immunotherapy, and chemotherapy drug sensitivity in glioma.

## 2 MATERIALS AND METHODS

### 2.1 Data sources and process

The workflow of this study is shown in Figure S1. The acquisition and preprocessing of data can be found in Method S1.

### 2.2 Identification of TRP channel clusters

Twenty TRPGs were collected from the GeneCards database. The TCGA, CGGA325, CGGA693, GSE16011, GSE108474, and GSE4412 were clustered using the unsupervised average linkage K‐means clustering analysis^24^, ^25^ and repeated 1000 times to ensure classification stability.^26^ Principal component analysis (PCA) was used to verify the clustering of the above gene expression profiles.

### 2.3 Construction of the MLTS prognostic model

As our previous study reported,^27^ we constructed the MLTS on the basis of ten classical machine learning algorithms and six multicenter glioma cohorts. The detailed process was provided in Method S1.

### 2.4 Intracranial homograft model and imaging

We injected a suspension of GL261‐Luc in PBS intracranially to establish mouse brain tumors. One week later, we performed bioluminescence imaging with a PerkinElmer IVIS Lumina 3 system to verify whether the homograft model was successful. See detailed information in Method S1.

### 2.5 Statistical analysis

All statistical analyses were performed by R software (version 4.0.2), procedure details are provided in Method S1.

Other bioinformatics methods and experimental methods are provided in Method S1.

## 3 RESULTS

### 3.1 Genomic and transcription characteristics of TRPGs

We collected 20 TRPGs from the GeneCards database, including *TRPA1*, *TRPC1*, *TRPC3*, *TRPC4*, *TRPC5*, *TRPC6*, *TRPC7*, *MCOLN1*, *MCOLN3*, *TRPM2*, *TRPM3*, *TRPM4*, *TRPM6*, *TRPM8*, *PKD2*, *PKD2L1*, *TRPV2*, *TRPV4*, *TRPV5*, and *TRPV6*. Next, we analyzed the frequency of single‐nucleotide variants (SNV) of these genes in pan‐cancer. We found SKCM, UCEC, and COAD had the highest mutation frequencies among all cancer types, while mutation frequencies in GBM and LGG were less than 11% (Figure S2A). Among the 1747 samples with mutated TRPGs, *TRPM6* had the highest mutation frequency (17%), followed by *TRPA1* (15%), and *TRPM3* (14%) (Figure S2B). Copy number variation (CNV) and mRNA expression levels were positively correlated in most cancer types, particularly in OV (Figure S2C). In contrast, DNA methylation levels were negatively correlated with mRNA expression levels in most cancers (Figure S2D). In this research, we focused on the effect of TRPGs in gliomas. The waterfall diagram showed 93 glioma patients had mutated TRPGs (Figure S2E). CNV was commonly observed for TRPGs. The CNV of *TRPV6* was found to be the highest, and that of *PKD2L1* was significantly low (Figure S2F).

**FIGURE 1:**
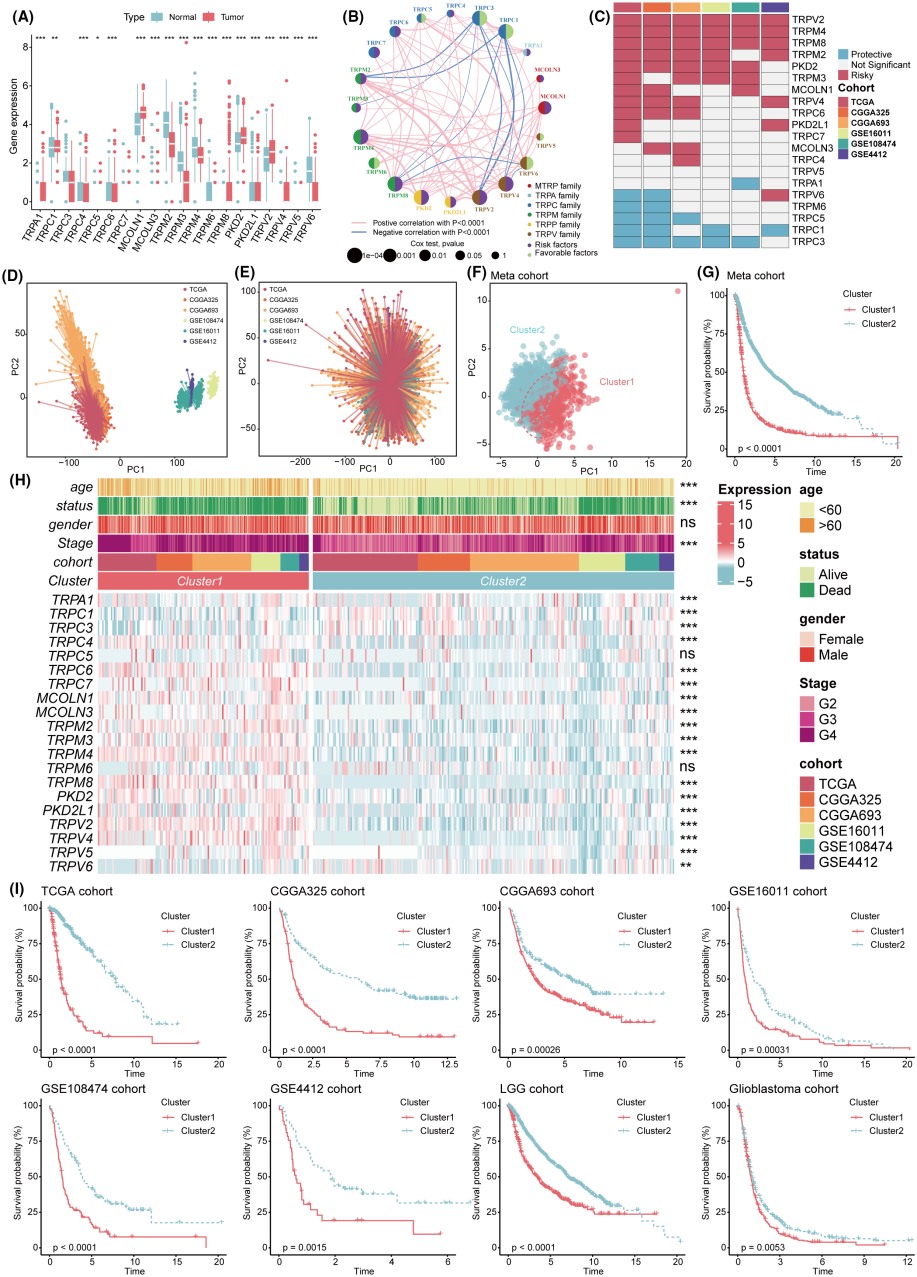
Identify two TRP clusters by unsupervised clustering. (A) Differences in mRNA expression of TRPGs in normal and tumor tissues in the TCGA cohort (*p < 0.05, **p < 0.01, ***p < 0.001). (B) The network showed the interaction between 20 TRPGs in gliomas in the TCGA cohort. (C) The heatmap showed the prognostic value of all TRPGs in six cohorts. (D, E) The PCA plots showed the expression baseline of the six cohorts before (D) and after (E) removing the batch effect. (F) PCA for the transcriptome profiles of TRP clusters in the meta‐cohort revealed a significant expression variation between the two clusters. (G) The Kaplan–Meier curves revealed significant prognostic differences between the two clusters in meta‐cohort. (H) The heatmap demonstrated the relationships between the two TRP clusters, clinicopathologic characteristics, and the expression variations of the 20 TRPGs. The top portion represented Fisher's precise test. The lower portion indicated the Wilcoxon rank‐sum test. ***p < 0.001, **p < 0.01, *p < 0.05, and ns stood for no statistical significance. Red in cells indicated high expression, while blue was connected with low expression. (I) The prognostic differences were observed in TCGA, CGGA325, CGGA693, GSE16011, GSE108474, GSE4412, LGG, and glioblastoma cohorts.

Expression levels of TRPGs were found to differ significantly between normal and glioma tissues, except for *TRPC3* and *TRPC7* (Figure 1A). Network maps illustrated the prognostic significance of TRPGs. Pearson's correlation analysis revealed the connection among them (Figure 1B).

### 3.2 Identification of TRP channel clusters using unsupervised clustering

To comprehensively understand the effect of TRP channels in gliomas, we applied univariate Cox analysis on 20 TRPGs in six independent glioma cohorts (Figure 1C). We considered TRPGs with *p* < 0.05 for at least three cohorts as consistent prognostic genes, including *TRPV2*, *TRPM4*, *TRPM8*, *TRPM2*, *PKD2*, *TRPM3*, *MCOLN1*, *TRPV4*, *TRPC6*, *TRPC5*, *TRPC1*, and *TRPC3*. Then, we combined the six cohorts into an integrated meta‐cohort. The expression baseline before (Figure 1D) and after (Figure 1E) removing the batch effect was visualized by PCA, indicating the batch effect had been effectively corrected. Considering the different expression patterns between low‐grade and higher‐grade gliomas, we extracted the LGG cohort and GBM cohorts from the meta‐cohort. Two TRP channel clusters (cluster1 and cluster2) were identified by k‐means clustering in all six cohorts. In addition, meta‐cohort, LGG cohort, and GBM cohorts also obtained the same results. The PCA plots confirmed the separation of two clusters based on the expression of TRPGs in all cohorts perfectly (Figure 1F, Figure S3A–H). Patients in cluster1 had higher expression levels of the TRPGs, older age, higher death rate, higher WHO grade, and fewer 1p/19q co‐deletion isocitrate dehydrogenase (IDH) mutations compared to those in cluster2 (Figure 1H, Figure S3I). We also analyzed the survival curves of patients in the two clusters and found that patients in cluster2 with low expression of TRPGs had a significant survival advantage. The same results were also shown in LGG cohort and GBM cohorts (Figure 1G,I). Based on the univariate and multivariate Cox analyses, cluster2 was a significant independent prognostic factor in almost all cohorts except GSE16011 and GSE4412 (Table S1).

### 3.3 Differences in the biological functions and genome characteristics of two TRP channel clusters

**FIGURE 2:**
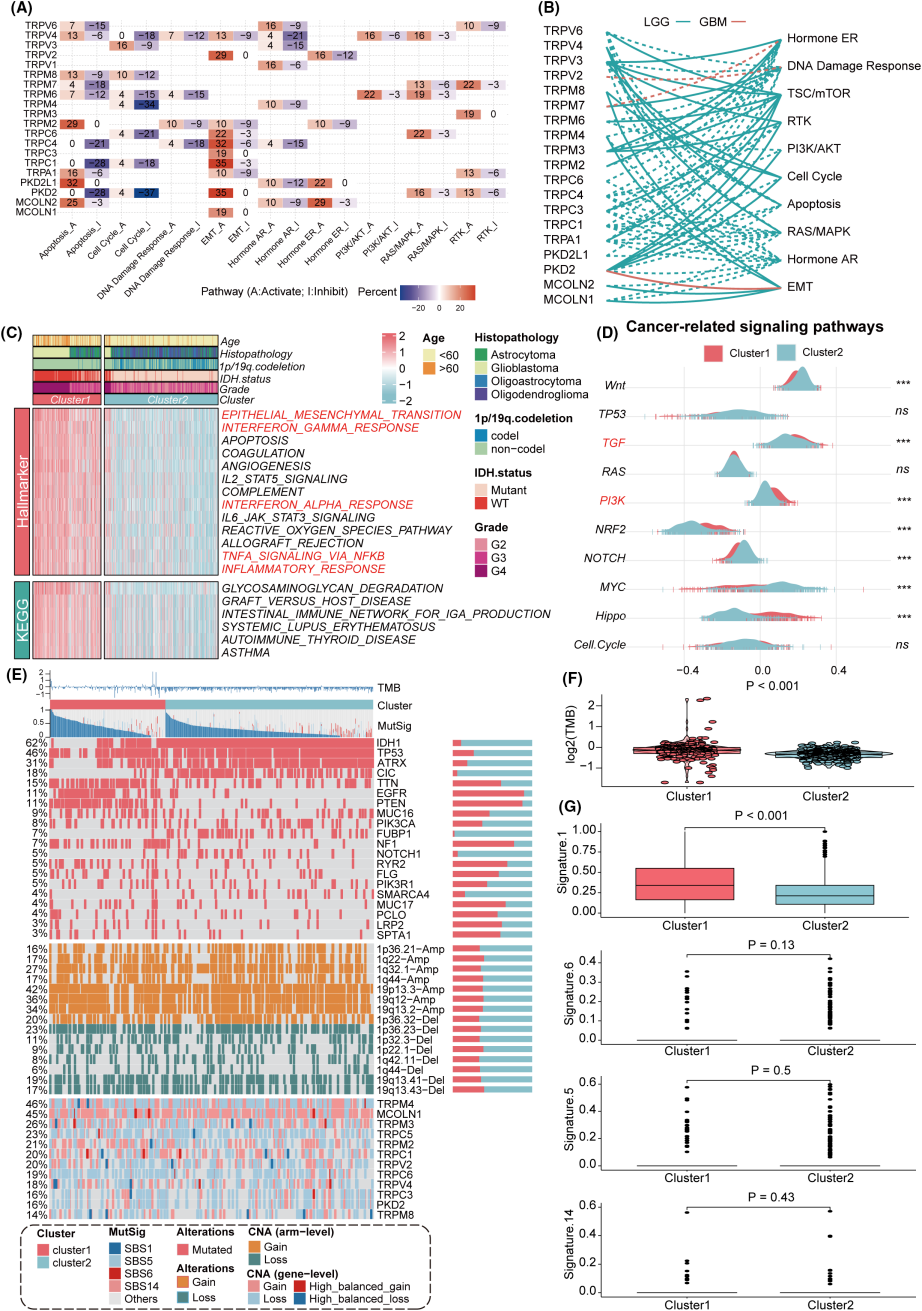
Investigations of functional pathways and genomic characteristics between TRP clusters. (A) The heatmap demonstrated the regulation of 20 TRPGs on broadly acknowledged cancer‐related pathways at the protein level. Only a function (inhibited or activated) was demonstrated in at least five cancer types. (B) The line connections illustrated the regulatory relationship between TRPGs and classical cancer pathways in the network. The solid line indicated activation, whereas the dotted line indicated inhibition. The green line indicated LGG, while the orange line indicated glioblastoma. (C) GSVA enrichment analyses in two TRP clusters illustrated the activation status of Hallmark and KEGG pathways in the TCGA cohort. (D) The ten vital broadly acknowledged cancer‐related signaling pathways between the two TRP clusters. Asterisks denoted *p*‐value (***p < 0.001 and ns was the abbreviation of no significance, Wilcoxon rank‐sum test). (E) The landscape of somatic mutations based on TRP clustering subtypes. Tumor mutation burden (TMB), the relative contribution of the four mutational signatures (SBS1, SBS5, SBS6, and SBS14), selected top‐mutated genes, and top‐broad‐level copy number alterations (*q*‐value 0.05), and selected TRPGs were displayed in ascending order from the top panel to the bottom. In the right stacked bar plots, the percentage of variation conversions was displayed. (F) TMB differences between the two clusters were revealed by the Wilcoxon rank‐sum test. (G) The four mutational signature variations between the two TRP phenotypes were revealed by the Wilcoxon rank‐sum test.

To mine for the underlying biological mechanisms of TRPGs, we investigated the roles of TRPGs in regulating recognized cancer‐related pathways in 32 cancer types through the TCPA database. In most cancers, TRPGs activated apoptosis and epithelial–mesenchymal transition (EMT) while inhibiting the cell cycle (Figure 2A). EMT and TSC/mTOR are also found to be activated in gliomas, aligned with the above results (Figure 2B). Subsequently, we investigated the biological functions of TRP channel clusters using GSVA. The results revealed that “epithelial–mesenchymal transition”, “interferon‐gamma response”, “interferon‐alpha response”, “TNFA signaling via NFKB”, and “inflammatory response” were activated in cluster1, suggesting that the TRP channels might affect the gliomas via immune‐related pathways (Figure 2C). We then compared the enrichment scores of the two clusters for ten notable cancer‐related pathways to explore the potential association between TRP channels and immune‐related pathways. In cluster1, TGF, PI3K, NRF2, and Hippo pathways were enriched, while in cluster2, the expressions of the Wnt, NOTCH, and MYC pathways were significantly elevated (Figure 2D).

We next investigated epigenetic variations in the identified TRP channel clusters based on CNVs, TMB, mutation signatures, and SNVs. The results revealed that cluster2 had relatively high mutation frequencies for *IDH1*, *ATRX*, and *CIC* among the top 20 highly mutated genes, while cluster1 had a high mutation frequency for *EGFR* and *PTEN*, which was considered as an immune escape‐related gene (Figure 2E). The CNVs of TRPGs are shown at the bottom of Figure 2E. Additionally, cluster1 exhibited a significantly higher TMB compared to cluster2 (Figure 2F). We analyzed four glioma‐related mutational signatures, namely SBS1 (age‐related), SBS5, SBS6 (DNA mismatch repair‐related), and SBS14. Only SBS1 showed a significant difference (Figure 2G).

### 3.4 TRP channels promote immunosuppressive microenvironment and immune escape in gliomas

Biological function analysis revealed that TRP channels might affect the tumor immune microenvironment (TIME), which was crucial for tumor growth, via immune‐related pathways and immune escape mutations in gliomas. Meanwhile, the activation of EMT leads to the accumulation of immunosuppressive cells in the tumor microenvironment.^28^ Thus, we investigated differences in TIME between the TRP channel clusters in the TCGA cohort. Considering TIME in GBM was significantly distinct from that of LGG, we conducted parallel analyses in the meta‐cohort, LGG cohort, and GBM cohort.

**FIGURE 3:**
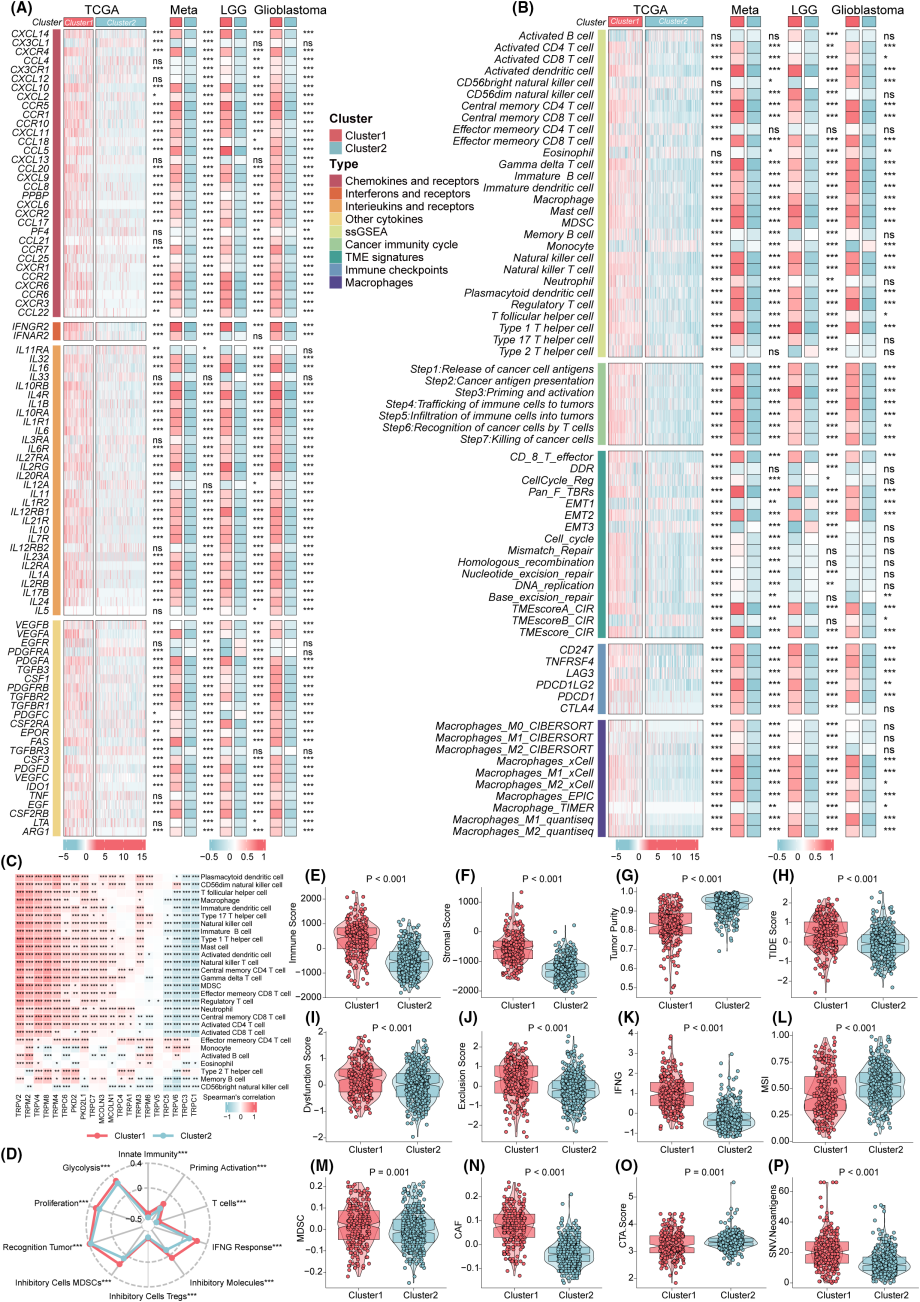
Alterations of immunomodulators and immune infiltration between two TRP clusters. (A) The heatmap displayed alterations in the mRNA expression levels of chemokines, interleukins, interferons, and other cytokines between the two TRP clusters in the TCGA cohort, meta‐cohort, LGG cohort, and glioblastoma cohort. (B) The heatmap displayed alterations in the immune infiltrating cell types, the enrichment levels of seven antitumor immune cycle steps, TIME signatures, immune checkpoints, and macrophages calculated by other algorithms between the two TRP clusters in the TCGA cohort, meta‐cohort, LGG cohort, and glioblastoma cohort. The asterisks denoted the *p*‐values (***p < 0.001, **p < 0.01, *p < 0.05, and ns was the shorthand for non‐significant for the Wilcoxon rank‐sum test). (C) The heatmap exhibited the correlation between mRNA expression levels of TRPGs and immune infiltrating cells. Asterisks denoted *p*‐value (*p < 0.05; **p < 0.01, ***p < 0.001). Blank cells represented no statistical significance of the correlation. (D) Immunogram radar plot displaying the variances of TIME signatures developed by Kobayashi. (E–P) Boxplots showed the differences in immune score (E), stromal score (F), tumor purity (G), TIDE (H), dysfunction score (I), exclusion score (J), IFN (K), MSI (L), MDSC (M), CAF (N), CTA score (O), and SNV neoantigens (P) between the TRP clusters.

We first conducted an investigation into the correlation between the clusters and multiple immunomodulators. Our findings indicated significant upregulation in the expression of immunosuppressive chemokines and receptors, such as *CXCL14*, *CCR10*, *CXCL16*, *CXCL9*, *CCL5*, *CCR2*, and *CCR5* in cluster1. Cluster1 showed higher levels of interleukins, interferons, receptors, and other cytokines in all grades of gliomas (Figure 3A). Subsequently, the single‐sample gene set enrichment analysis (ssGSEA) algorithm was employed to evaluate levels of immune cell infiltration. The results revealed that cluster2 exhibited higher levels of eosinophils and monocytes, whereas cluster1 displayed elevated levels of immunosuppressive cells, including myeloid‐derived suppressor cells, regulatory T cells, and macrophages. Our results were stabilized by calculating the abundance of macrophages, which are among the most abundant immune cells in gliomas, via five other algorithms (Figure 3B). We used the ssGSEA algorithm to calculate the scores of seven immune cycle steps representing antitumor immune response events. The results indicated that cluster1 exhibited higher scores in all seven steps (Figure 3B). Moreover, cluster1 demonstrated higher expression levels of six immune checkpoint genes, including *CD274*, *CD247*, *TNFRSF4*, *PDCD1LG2*, *PDCD1*, and *CTLA‐4*, which have the potential to facilitate immune escape and serve as promising targets for immunotherapy (Figure 3B). The TRP channel clusters were further distinguished based on different TIME‐related characteristics (Figure 3B). We obtained the same results in meta, LGG, and GBM cohorts.

Correlation analysis showed that expression levels of *TRPV2*, *TRPM2*, *TRPV4*, and *TRPM8* were positively correlated with most immune cell types, whereas the opposite was observed for *TRPC1*, *TRPC3*, and *TRPV6* (Figure 3C). Additionally, in the analysis of TIME signatures developed by Kobayashi, cluster1 had higher levels of innate immunity, priming activation, T cells, IFN‐γ response, Treg, MDSC, tumor recognition, proliferation, and glycolysis (Figure 3D). Using the tumor immune dysfunction and exclusion (TIDE) algorithm, our analysis revealed cluster1 exhibited higher TIDE scores, exclusion scores, dysfunction scores, immune scores, stromal scores, IFNG, MDSC, SNV.neoantigens, and CAFs while displaying lower tumor purity scores and microsatellite instability (MSI) (Figure 3E–P). Notably, although patients in cluster1 had high levels of immune infiltration, the presence of immunosuppressive cells, high concentrations of immunoinhibitory cytokines, high expression of immune checkpoints, high TIDE scores, and high levels of CAF may cause immune escape in cluster1, consistent with the poor prognosis of cluster1 patients.

### 3.5 Single‐cell RNA sequencing analysis confirmed the association between TRP channels and macrophages

**FIGURE 4:**
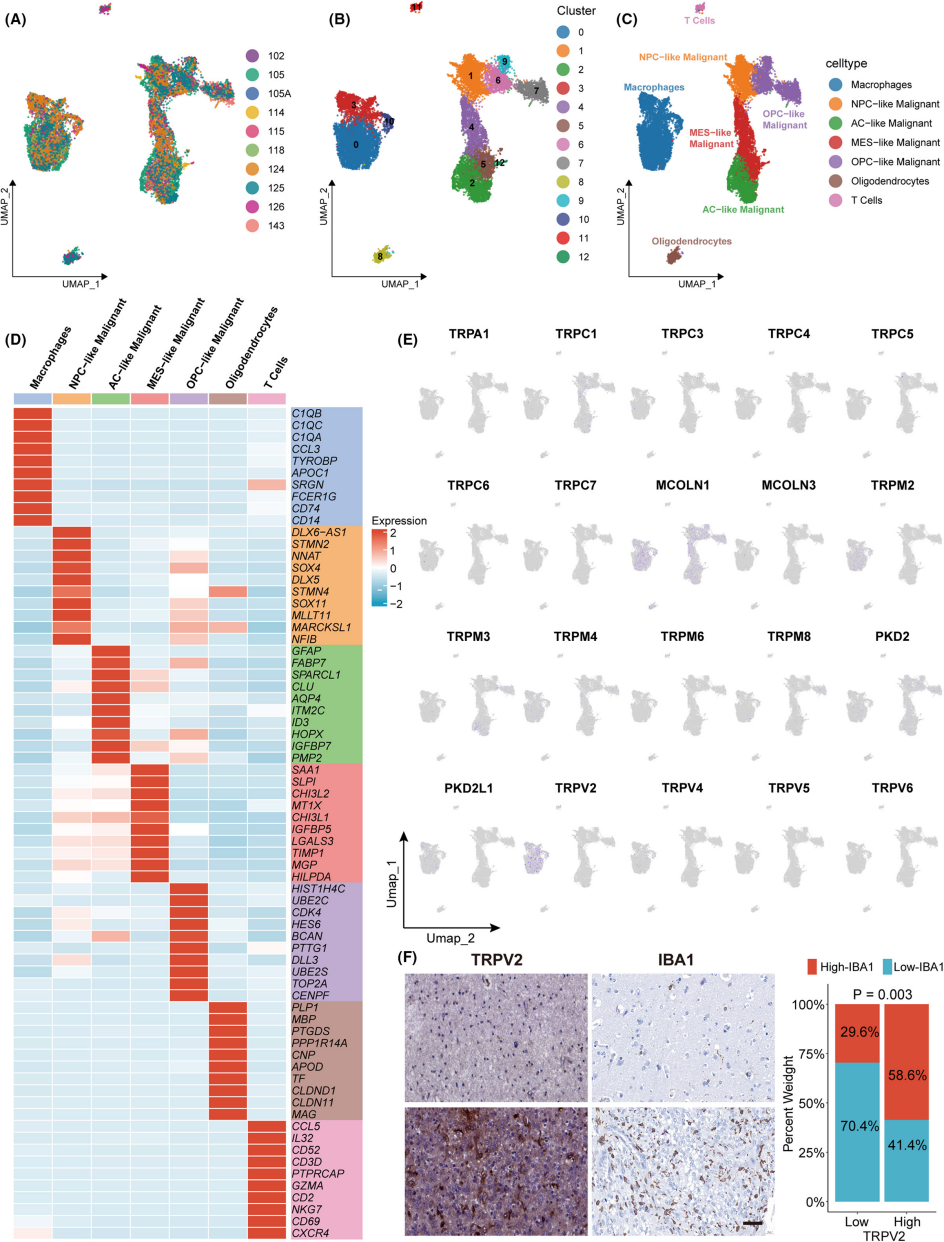
Analysis of single‐cell RNA sequencing data to investigate the expression of TRPGs in different cell types. (A–C) UMAP plots of 10 glioma samples after removing batch effect (A), 13 cell clusters (B), and 7 cell types (C). (D) The heatmap showed the markers of 7 cell types. (E) UMAP plots of the expression of 20 TRPGs. (F) Representative IHC staining images and semi‐quantitative analyses of TRPV2 and IBA1 in human glioma tissues (*n* = 56, scale bars = 50 μm).

Based on the results above, the TRP channel might be a glioma risk factor, associated with the immunosuppressive microenvironment. To investigate its immunological implications, we downloaded single‐cell RNA sequencing data GSE131928 and integrated 16,201 cells from 10 glioma samples. First, we removed the batch effect of 10 samples (Figure 4A). Dimensionality reduction and clustering were applied to the data. Then, according to the previous study,^29^ seven superclasses of cell types were identified: oligodendrocytes, macrophages, T cells, AC‐like malignant cells, OPC‐like malignant cells, MES‐like malignant cells, and NPC‐like malignant cells (Figure 4B,C). Markers of seven cell types were shown in the heatmap (Figure 4D). Based on the results above, we focused on the immune cells. *TRPM2*, *TRPM4*, *PKD2L1*, *TRPV2*, and *TRPV4* were specifically expressed in macrophages, which aligned with the results of the immune infiltration correlation analysis of bulk RNA‐seq data (Figure 4E). *TRPV2* had the highest positive correlation with immune cells in bulk RNA‐seq analysis. It also had the highest expression level in macrophages in single‐cell analysis. Furthermore, the univariate Cox analysis showed that *TRPV2* was a risk factor in all six cohorts. Therefore, we selected *TRPV2* for further investigation. Subsequently, we collected eight additional glioma scRNA‐seq datasets from TISCH2 database, including GSE102130, GSE103224, GSE141383, GSE141460, GSE141982, GSE148842, GSE70630, and GSE89567 to investigate *TRPV2* expression in different cell types (Figure S4A,B). *TRPV2* was predominantly expressed in monocytes and macrophages (Figure S4C). We also performed IHC staining of 56 glioma tissues to confirm the correlation between the levels of *TRPV2* and the macrophages (Figure 4F).

### 3.6 TRPV2 activation affects tumor progression in glioma

**FIGURE 5:**
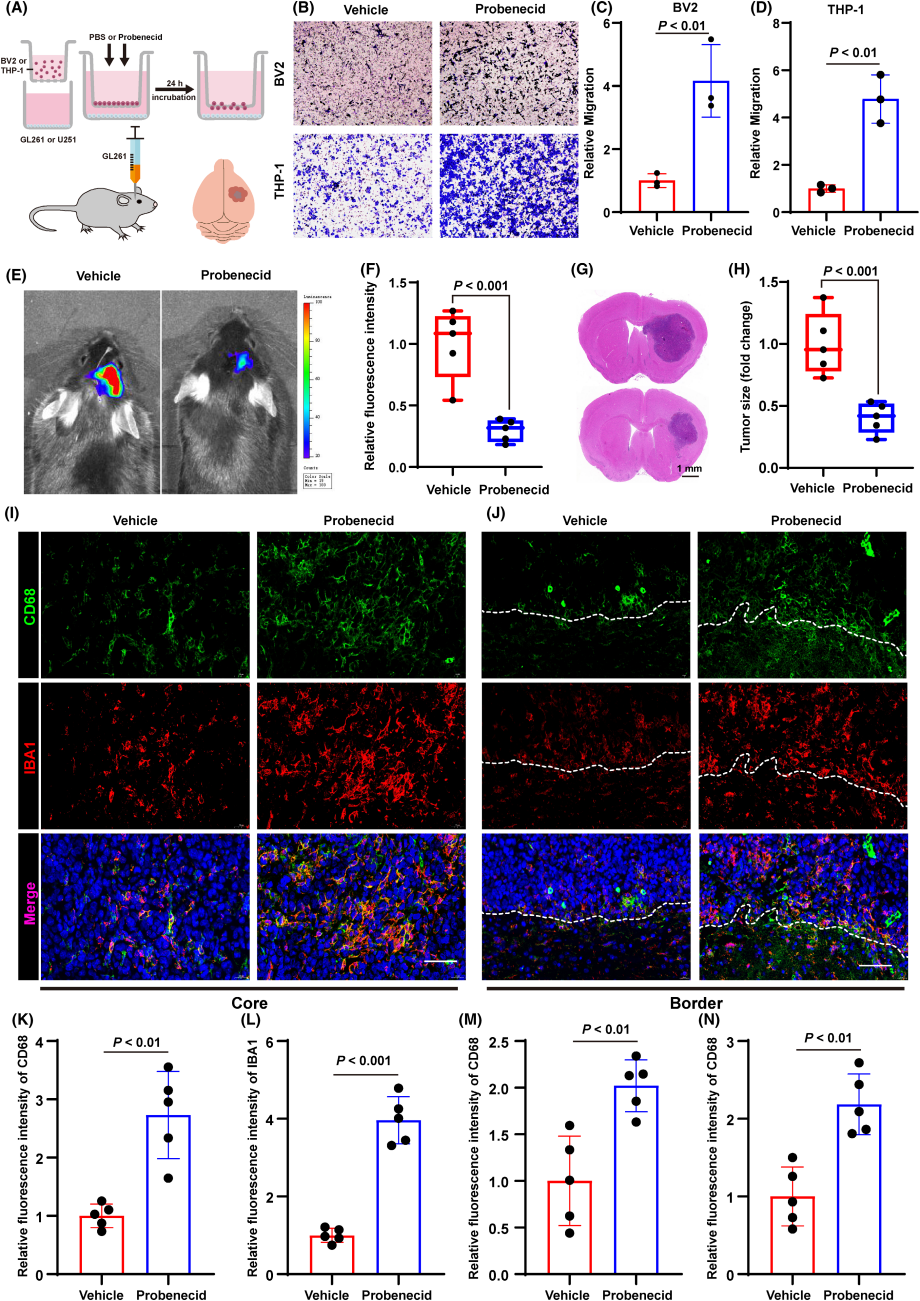
TRPV2 activation is associated with tumor progression in gliomas. (A) Schematic diagram for the experiment. (B–D) Transwell assay of BV2 and THP‐1 cells co‐cultured with glioma cells (GL261 and U251), *n* = 3. (E, F) Representative IVIS imaging of the tumor‐bearing mice, *n* = 5. (G, H) HE staining of brain tissues of the tumor‐bearing mice, scale bar = 1 mm, *n* = 5. (I–N) Representative Immunofluorescence images showing the expression levels of CD68 and IBA1 in the core and border areas of gliomas, scale bar = 50 μm, *n* = 5. All values represent SD ± mean.

Based on the results presented in the previous sections, we reasoned that the TRP channel might affect the TIME in gliomas. It has been reported that *TRPV2* mediated the migration and the degranulation of macrophage.^21^ Therefore, we aimed to investigate the immunological implications of *TRPV2* in gliomas. First, we performed a transwell assay by co‐culturing BV2 with GL261 cells and THP1 with U251 cells, respectively, to evaluate the migration ability of microglia/macrophages (Figure 5A). Glioma cells (GL261/U251) were found to induce the migration of microglia/macrophages (BV2/THP‐1). Probenecid, a TRPV2 agonist, promoted the migration of BV2 and THP‐1 cells (Figure 5B–D). Furthermore, we created an intracranial homograft model in mice using GL261 cells and monitored the size of the tumor using the Perkin‐Elmer IVIS Lumina 3 system. The result showed that the fluorescence signal in the TRPV2 agonist treatment group was lower than that in the vehicle‐treated group (Figure 5E,F). HE staining demonstrated the same results (Figure 5G,H). Subsequently, we performed IF to investigate whether probenecid affected microglial/macrophage infiltration. Our results showed that, compared to the vehicle group, probenecid treatment increased the number of CD68‐positive cells and IBA1‐positive cells in the tumor core area and the border area (Figure 5I–N).

### 3.7 Construction of the machine learning‐based TRP channel signature

**FIGURE 6:**
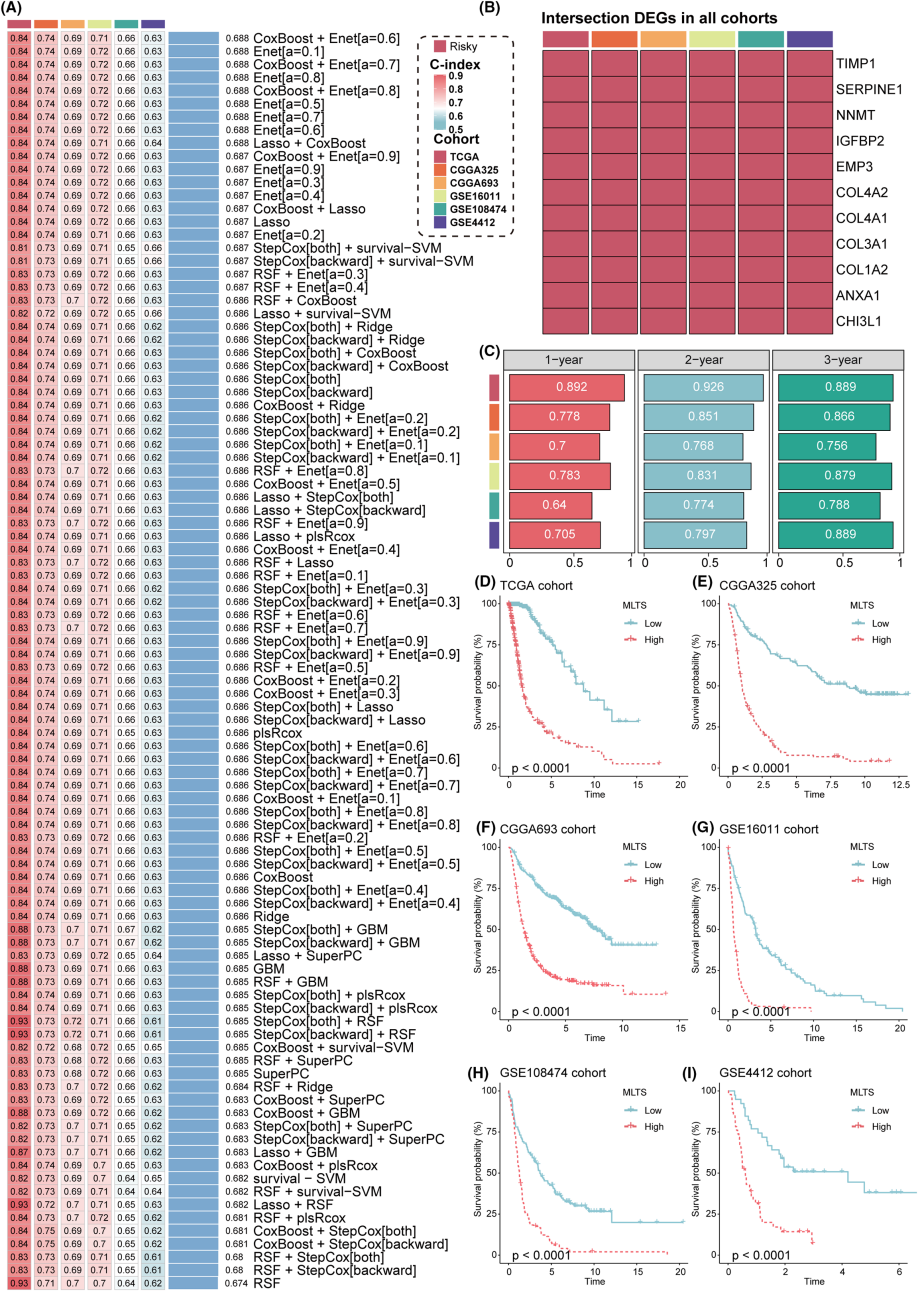
Generation of a signature of TRP channels based on machine learning. (A) C‐indices of 96 machine learning algorithms in eight glioma cohorts. (B) This heatmap demonstrated the prognostic value of the 11 prognostic TRP‐related DEGs specific to gliomas in six cohorts. (C) Time‐dependent ROC analysis for predicting OS at 1, 2, and 3 years in TCGA‐Gliomas, CGGA325, CGGA693, GSE16011, GSE108474, and GSE4412. (D–I) OS Kaplan–Meier survival curves between glioma patients with high and low MLCS in the TCGA (D), CGGA325 (E), CGGA693 (F), GSE16011 (G), GSE108474 (H), and GSE4412 (I) cohorts.

To further explore the clinical significance of the TRP channels in gliomas, the “limma” R package was performed to identify the TRP‐related genes specific to gliomas. We screened out the differently expressed genes (DEGs) between cluster1 and cluster2 with adj. *p* < 0.05 and |logFC| > 1.5 as cutoffs in six cohorts. The DEGs upregulated or downregulated consistently in all six cohorts were enrolled for further analysis. The univariate Cox analysis was also applied to identify 11 prognostic TRP‐related genes specific to gliomas, including TIMP1, SERPINE1, NNMT, IGFBP2, EMP3, COL4A2, COL4A1, COL3A1, COL1A2, ANXA1, and CHI3L1 (Figure 6B). Interestingly, all these DEGs were risk factors for gliomas. Subsequently, we applied these 11 DEGs and 12 consistent prognostic TRP genes to construct a machine learning‐based TRP channel signature (MLTS). In the TCGA training cohort, we employed 96 combinations based on 10 machine learning algorithms with tenfold cross‐validation and calculated the average C‐index of each model in the seven testing cohorts to evaluate the prognostic prediction ability of all models (Table S2). The algorithm involving the Coxboost and Enet (a = 0.6) exhibited the highest average C‐index and was therefore considered the optimal model among all 96 algorithms (Figure 6A). The correlation analysis confirmed the strong association between MLTS, TRPGS, and the 11 prognostic TRP‐related genes specific to gliomas (Figure S5A–F).

### 3.8 Robust prognostic value of MLTS in gliomas

**FIGURE 7:**
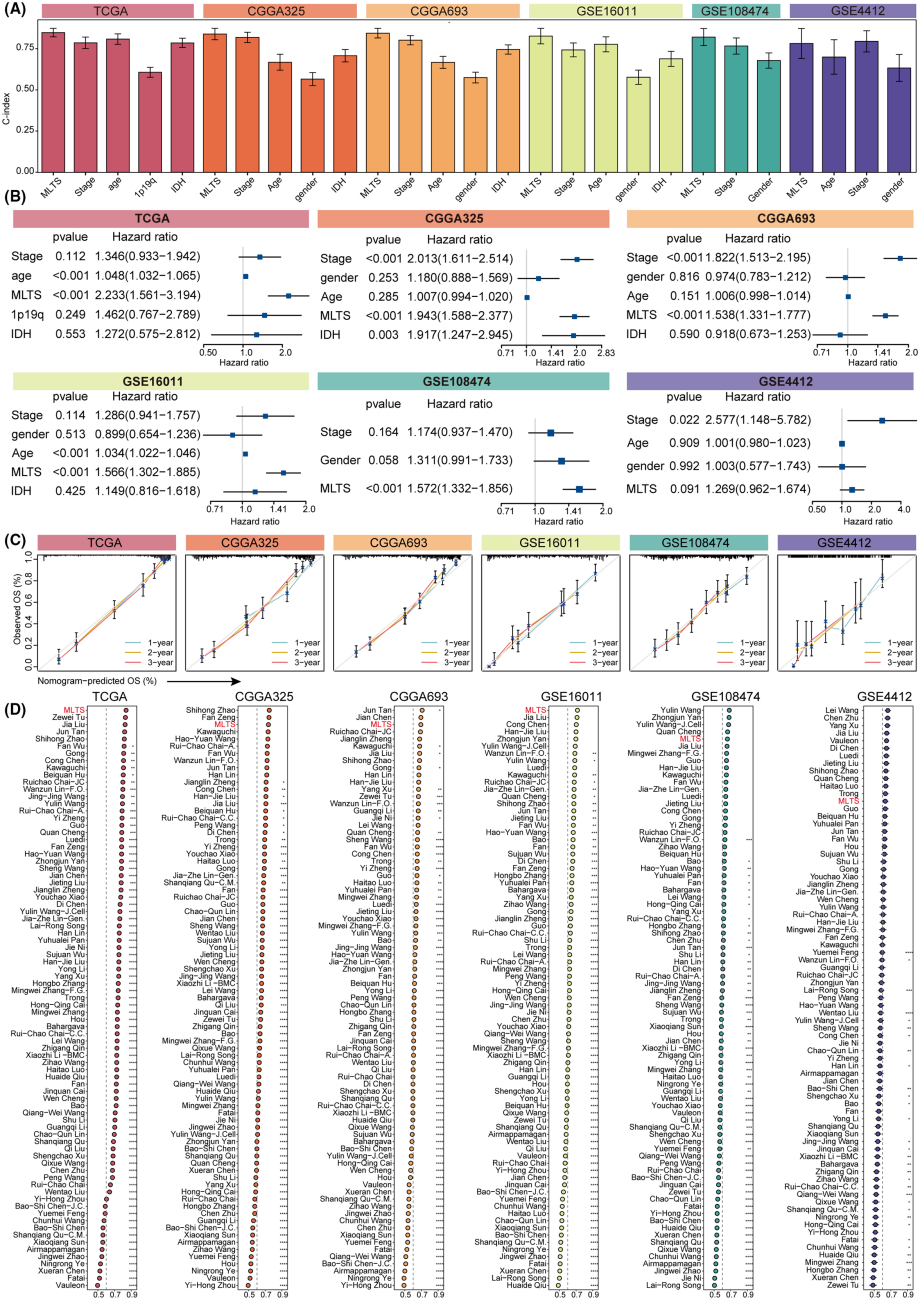
The robust and stable predictive ability of MLTS in Gliomas. (A) The performance of MLTS was compared with other clinical traits in predicting prognosis. (B) Multivariate Cox regression analysis of overall survival (OS) in the TCGA‐Gliomas, CGGA325, CGGA693, GSE16011, GSE108474, and GS4412. (C) Plots depicted the calibration of nomograms in the TCGA‐Gliomas, CGGA325, CGGA693, GSE16011, GSE108474, and GSE4412. (D) C‐indices of MLTS and 80 published signatures in TCGA‐Gliomas, CGGA325, CGGA693, GSE16011, GSE108474, and MLTS.

To evaluate the prognostic value of MLTS, we divided the patients with gliomas into high‐ and low‐MLTS groups in each of the six cohorts. The survival outcomes of patients in the high‐MLTS group were shorter than those in the low‐MLTS group in TCGA, CGGA325, CGGA693, GSE16011, GSE108474, and GSE4412 cohorts (Figure 6D–I). The robust prognostic predictive power of MLTS was confirmed by the ROC curve of 1‐, 2‐, and 3‐year OS and the mean C‐index of MLTS in TCGA, CGGA325, CGGA693, GSE16011, GSE108474, and GSE4412 cohorts (Figure 6A,C). Besides, we conducted the multivariate Cox regression analysis to assess the independence of MLTS, common clinical traits, and molecular features. MLTS was identified as an important independent risk factor for gliomas in TCGA, CGGA325, CGGA693, GSE16011, and GSE108474 cohorts (Figure 7B). Due to the small sample size, the statistical significance of MLTS could not be reliably determined in the GSE4412 cohort. The calibration curves further confirmed the predictive accuracy of the MLTS signature (Figure 7C).

### 3.9 Favorable predictive performance of MLTS

Classical clinical and molecular characteristics were traditionally used to evaluate outcomes. Therefore, we compared the MLTS with these clinical characteristics, suggesting that the MLTS model showed superior performance in predicting prognosis (Figure 7A). Increasingly, gene expression‐based biomarkers have been used as prognostic biomarkers for cancer. We compared the predictive ability of MLTS with that of 80 other mRNA signatures from previously published studies. MLTS ranked first in TCGA and GSE16011, third in CGGA325 and CGGA693, and fifth in GSE108474 (Figure 7D).

### 3.10 MLTS predicts immunotherapy response and sensitivity to chemotherapeutic drugs in gliomas

**FIGURE 8:**
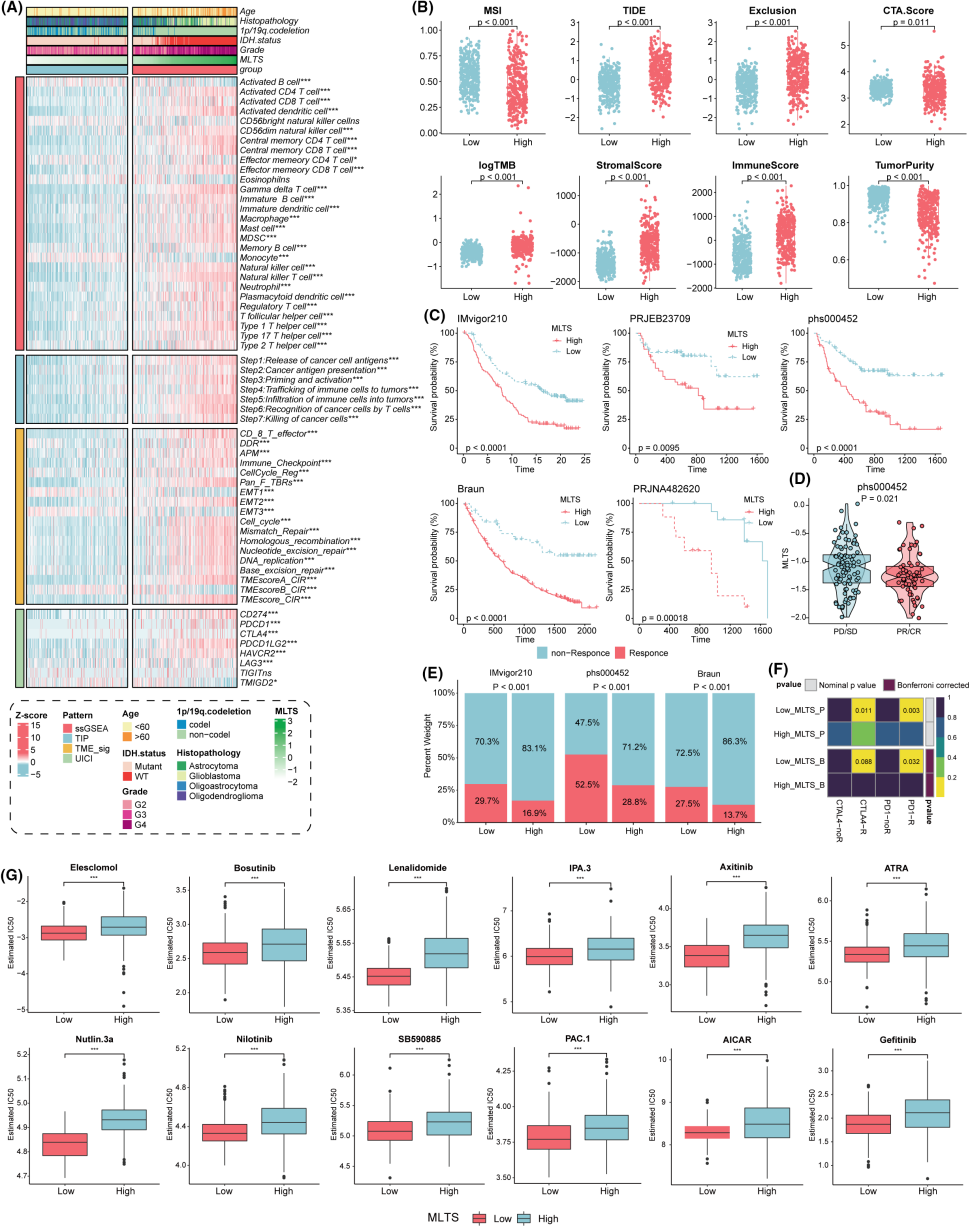
MLTS can predict immunotherapy response and drug sensitivity of glioma. (A) This complex heatmap displayed the tumor immune microenvironment profile in the TCGA‐Gliomas cohort, including immune infiltrating cell types, immune‐related pathways, the enrichment levels of seven antitumor immune cycle steps, and immune checkpoints. Annotated clinicopathologic characteristics are at the top of the heatmap. (B) Box plots showed significant differences in MSI, TIDE, exclusion score, CTA. Score, TMB, stromal score, immune score, and tumor purity between high‐ and low‐MLTS groups. (C) Kaplan–Meier survival curves of OS in glioma patients with high and low MLTS from the IMvigor210, PRJEB23709, phs000452, Braun, and PRJNA482620 cohorts. (D) Box plot revealed the MLTS of glioma patients with different immunotherapy responses in the phs000452 cohort. (E) Stack histogram showed the ratio of immunotherapy reactivity between high‐ and low‐MLTS groups in IMvigor210, phs000452, and Braun cohorts. (F) Immunotherapy response (anti‐PD‐1 and anti‐CTLA‐4) and the MLTS group based on submap analysis. (G) Difference of IC50 in common clinical chemotherapy drugs between high‐ and low‐MLTS groups (Wilcoxon test, *p < 0.05, ***p < 0.001).

For predicting the immunotherapy response, we next examined the immune landscape in high‐ and low‐MLTS groups. As shown in Figure 8A, immune cell infiltration analysis based on the ssGSEA algorithm revealed that patients in the high‐MLTS group had stronger immune cell infiltration. Moreover, the high‐MLTS group showed upregulation of genes in immune‐related pathways, presenting an inflammatory TIME phenotype (Figure 8A). MLTS was also positively correlated with the seven stages of the immune cycle in all cohorts (Figure 8A). Analysis of immune checkpoints further showed that multiple immune checkpoint genes were highly expressed in the high‐MLTS group, including *CD274*, *PDCD1*, *CTLA4*, *PDCD1LG2*, *HAVCR2*, *LAG3*, and *TMIGD2* (Figure 8A). The stromal and immune scores were higher in the high‐MLTS group, whereas tumor purity was lower (Figure 8B). Notably, the TIDE, exclusion, and TMB scores were significantly higher in the high‐MLTS group, whereas MSI was higher in the low‐MLTS group, suggesting that patients with low MLTS might benefit more from immune checkpoint inhibitors (Figure 8B).

Patients with low MLTS had lower TIDE and higher MSI scores, suggesting that the low‐MLTS group might benefit more from immunotherapy. Therefore, we explored predictive value of MLTS for immunotherapy responses. The IMvigor210, PRJEB23709, phs000452, Braun, and PRJNA482620 cohorts were enrolled and divided into high‐ and low‐MLTS groups. Survival curve analysis showed that the low‐MLTS group had a better prognosis in the IMvigor210, PRJEB23709, phs000452, Braun, and PRJNA482620 cohorts (Figure 8C). Simultaneously, the low‐MLTS groups in the IMvigor210, phs000452, and Braun cohorts showed more effective anti‐PD‐L1 immunotherapy responses (Figure 8D,E). Furthermore, we used the submap algorithm to calculate the ability of MLTS to predict immunotherapy and found that patients in the low‐MLTS group benefited more from anti‐PD1 and anti‐CTLA4 therapy (Figure 8F).

Moreover, we analyzed the differences in semi‐maximum inhibitor concentration (IC50) between high‐ and low‐MLTS and glioma chemotherapeutic drugs. The results showed that patients with low MLTS had a lower IC50 in response to multiple chemotherapeutic drugs (Elesclomol, Bosutinib, Lenalidomide, IPA.3, Axitinib, ATRA, Nutlin.3a, Nilotinib, SB590885, PAC.1, AICAR, Gefitinib), suggesting that patients with low MLTS were more sensitive to chemotherapy (Figure 8G).

## 4 DISCUSSION

In this study, we collected 20 TRPGs and classified two TRP channel clusters in patients with glioma using a k‐means clustering method. Biological function analysis of the TRP channels revealed that most TRPGs consistently activated the immune‐related pathways and EMT at the protein level. The GSVA analysis confirmed our conclusions above at the mRNA level. We found that the EMT, IFNG, and TGFB pathways were highly enriched in cluster1 patients with high expression levels of TRPGs. The TGFB pathway was identified as an essential activator for EMT.^30^, ^31^ Furthermore, cluster1 patients also had a higher enrichment score for the PI3K pathway, implying that TRP channel activation could remodel the immunosuppressive microenvironment via the EMT pathway. Subsequently, we analyzed the genome alterations between the two clusters. Compared to cluster2, patients in cluster1 had more mutations in immune escape‐related genes, such as *EGFR* and *PTEN*. Furthermore, a higher level of TMB was observed in cluster1, suggesting that patients in cluster1 had stronger immune escape capability as well as higher immunogenicity, producing more tumor neoantigens. However, increased expression of immune checkpoints in cluster1 resulted in blocked antigen presentation. According to a previous immunoediting theory,^32^ these results constitute an intrinsic immune escape mechanism in cluster1 patients with high TRPGs expression.

To explore the detailed mechanism of the effects of the TRP channel on the immune microenvironment, we conducted immune cell infiltration analyses and obtained similar results. Almost all immune cells were enriched in cluster1, including T cells, macrophages, MDSCs, and Treg cells. Gliomas have a unique immune microenvironment predominated by macrophages and MDSCs. Therefore, patients in cluster1 had more activated immunosuppressive components, presenting a stronger immunosuppressive microenvironment. *CXCL14*, *CCR10*, *CXCL16*, *CXCL9*, *CCL5*, *CCR2*, and *CCR5*, considered as immunosuppressive cytokines and chemokines, were also highly expressed in cluster1. Furthermore, a higher CAF score in cluster1 means a higher level of fibrosis. These results might promote the migration of immunosuppressive cells to malignant cells, leading to the dysfunction of T cells, consistent with high TIDE scores and exclusion scores. Consequently, we hypothesized that high levels of immunosuppressive cells, high concentrations of immunosuppressive factors, and high levels of fibrosis constitute the extrinsic immune escape mechanisms in cluster1 patients with high TRPGs expression.

To validate the specific role of TRP channels in the glioma immune microenvironment, we investigated the expression distribution of TRPGs in different cell types by analyzing single‐cell sequencing data. We selected TRPV2, which was identified as the strongest immune‐related gene and was specifically expressed in macrophages.^21^ We co‐cultured glioma cells and macrophages in vitro and found that probenecid (a TRPV2 agonist) could promote the migration of macrophages to malignant cells. An intracranial homograft model was then developed. We showed that probenecid promoted the migration of macrophages toward tumor cells, which is consistent with the results above. Interestingly, a relatively significant prognostic advantage was observed in the treatment group, suggesting that TRPV2 exerts an antitumor effect. This conclusion did not contradict the results of bioinformatics analysis. Univariate COX regression analysis demonstrated that most TRPGs and TRPV2 were prognostic risk factors for gliomas. However, this might be the result of TRPV2 activation for its antitumor effects during the tumor progression. Furthermore, TRPV2 that was mainly expressed in macrophages was activated during tumor progression to attract immunosuppressive components, such as macrophages, toward malignant cells for phagocytosis. However, the activated antitumor components were insufficient to initiate an antitumor immune response by overcoming immune escape and immunosuppressive microenvironments, explaining the worse prognosis of patients in cluster1.

Considering the critical role of the TRPGs in gliomas,^17^ we developed an artificial intelligence‐driven TRP channel signature to predict the prognosis of patients with gliomas and provide new strategies for treatment. Similar to the approach used in previous studies,^27^ we combined ten classical machine learning algorithms into 96 combinations. Subsequently, we conducted all combinations in the training cohort and five validation cohorts. We selected the model with the highest average c‐index to construct a machine learning‐based TRP channel signature named MLTS. Compared to common clinical variables and published biomarkers, MLTS demonstrated a favorable prognostic predictive ability. Furthermore, patients in the high‐MLTS group exhibited stronger immune escape and an immunosuppressive microenvironment. Therefore, the MLTS could be used to predict immunotherapy responses. Our study found that patients in the low‐MLTS group had a higher MSI score and a lower TIDE score, which were related to a more inefficient activation of the immunosuppressive microenvironment. We validated these results in cohorts in which patients received immune checkpoint inhibitor treatment. Notably, the low‐MLTS group responded better in the glioma immunotherapy cohort. Subsequently, drug sensitivity analysis demonstrated that the low‐MLTS group was more sensitive to chemotherapeutic agents. These results suggest that MLTS was an outstanding biomarker for predicting prognosis and immunotherapy response in gliomas.

In conclusion, based on bioinformatics analysis, we elucidated the role of TRP channels in gliomas and revealed their potential immune escape mechanisms. TRPV2 was demonstrated to promote macrophage migration toward malignant cells and improve glioma prognosis in in vitro and in vivo experiments. We constructed a stable and robust model (MLTS) for predicting glioma prognosis, immunotherapy response, and chemotherapy drug sensitivity based on multicenter integrated analysis and machine learning algorithms. MLTS is a promising tool to develop individualized glioma treatment strategies.

## AUTHOR CONTRIBUTIONS

Yuntao Li and Shi Feng analyzed the data. Xiaoxing Xiong and Qianxue Chen designed this study. Yuntao Li and Yonggang Zhang wrote the article and conducted cellular experiments. All authors revised the manuscript.

## FUNDING INFORMATION

This work was supported by the National Natural Science Foundation of China (82072764) to Qianxue Chen, the Fundamental Research Funds for the Central Universities (2042022kf1216) to Xiaoxing Xiong, and the Zhejiang Provincial Natural Science Foundation of China (No.LQ19H030001) to Ying Xing.

## CONFLICT OF INTEREST STATEMENT

It is declared that none of the authors have any conflicts of interest.

## Supporting information


Appendix S1

Table S1

Table S2

## Data Availability

Data supporting the findings of this study are available upon reasonable request from the corresponding author.
