# Supplementary material for: Multicenter integration analysis of TRP channels revealed potential mechanisms of immunosuppressive microenvironment activation and identified a machine learning‐derived signature for improving outcomes in gliomas
Source: CNS Neurosci Ther. 2024 Jul 1;30(7):e14816. doi: 10.1111/cns.14816 (PMC11215471; doi:10.1111/cns.14816)
Supplement: Supplementary file 1 — Appendix S1 [file CNS-30-e14816-s002.docx]

**Supplementary methods**

**Acquisition and preprocessing of data**

All data used in this study came from public databases. TCGA-LGG, TCGA-glioblastoma and GTEx-Brian transcriptome data obtained from the UCSC database (https://xenabrowser.net/datapage/). We downloaded RNA sequencing data and corresponding clinicopathological information from the CGGA website (https://www.cgga.org.cn). Through Gliovis website (http://gliovis.bioinfo.cnio.es/) we downloaded the GSE16011, GSE108474, and GSE4412 glioma cohorts. RNA sequencing data was converted into millions per kilobase (TPM) format. Microarray data from the Affymetrix platform was normalized and background corrected using the "affy" R software package. From cbioportal database (http://www.cbioportal.org/) we retrieval and searched the clinical phenotype corresponding to transcriptome data. Batch effects were corrected using the "sva" package in R software ^1^. A total of 2002 patients were included after removing samples without survival information. We obtained TCGA-LGG and TCGA-glioblastoma genome mutation data, including Single Nucleotide Variation (SNV) and Copy Number Variation (CNV), from the UCSC database. We used the software package "maftools" to analyze the SNV data and GISTIC2.0 to detect the amplification and deletion of CNV. RPPA proteomic data was used to calculate activation and inhibition scores for 10 recognized cancer-related pathways in 32 cancers using the TCPA database. On the GSCAlite website (http://bioinfo.life.hust.edu.cn/web/GSCALite/) we conducted parts of the TCGA pan cancer analysis and RPPA data analysis ^2^.

**Analysis of single cell RNA sequencing**

We analyzed single-cell RNA sequencing data of human glioma tissues (n=10). The GSE131928 was downloaded from the GEO database (<https://www.ncbi.nlm.nih.gov/geo/>). Cell clustering and dimension reduction were performed via the “Seurat” package^3^ We used the function “RunPCA” to conducted the principal component analysis. “FindNeighbors” was used to construct unsupervised clustering of cells. The most mutated cells were combined using the function “FindClusters.” The dimensionality reduction was conducted by the function “UMAP”. Referring to previous posts^3^, the annotation of different cell types was performed. Subsequently, we showed the expression of TRPGs in different cell types. Other glioma single-cell sequencing data was obtained from TISCH2 website (<http://tisch.comp-genomics.org/>), including GSE102130, GSE103224, GSE141383, GSE141460, GSE141982, GSE148842, GSE70630, and GSE89567^4^. Then, we evaluated the expression of TRPV2 in stromal cells, immune cells, and malignant cells using these datasets.

**Evaluation of tumor immune microenvironment**

The ESTIMATE algorithm was used to estimate tumor purity, immune score, and stromal score^5^. In addition, ssGSEA algorithm^6^, CIBERSORT algorithm^7^, XCELL algorithm^8^, TIMER algorithm^9^, QUANTISEQ algorithm^10^ and MCPCOUNTER algorithm^11^ were used to calculate the infiltration level of immune cells in gliomas. We obtained a list of 100 immunomodulatory genes from previous studies, including chemokine receptors, interleukin and interferon^6, 12^. Online website TIDE (http://tide.dfci.harvard.edu/) was applied to the analysis of tumor immune dysfunction and exclusion (TIDE) score. TIDE scores in highly cytotoxic T cell (CTL) malignancies were correlated with T cell dysfunction and T cell immune factor exclusion^13^. We downloaded the TME-related trait gene set using the IBOR R package^14^ and examined the scores of these data sets in gliomas using the ssGSEA algorithm. We downloaded the tumor immune cycle the seven steps of genetic data through tumor immune tracking website (<http://biocc.hrbmu.edu.cn/TIP/index.jsp>)^15^ and quantified them using ssGSEA.

**Analysis of pathway enrichment**

First, we investigated the correlation between TRP channels and cancer-related pathways in 32 cancers. Pathway scores were calculated by adding the difference between the protein levels of all positive and negative regulatory components. Patients were divided into two groups based on median protein levels of TRPGs^16^. When the high expression group scored higher than the low expression group, it indicated that the pathway was active^17^. We collected gene sets for these 10 cancer-related pathways from previous studies in order to detect the expression of cancer pathways at the transcriptome level. In addition, we scored them using the ssGSEA algorithm. We downloaded "h.all.v7.4.symbols" "and "c2.cp.kegg.v7.4.symbols" from MsigDB database (https://www.gsea-msigdb.org/gsea/msigdb), and apply gene set variation analysis (GSVA) program to explored the biological differences between two subtypes of TRPGs^18^.

**Development of the TRP Prognostic Model**

We converted the gene expression profiles of all cohorts into z-scores to enhance the comparability between different cohorts as previously reported^19-21^. We used cohorts with survival information, including TCGA-Gliomas, CGGA325, CGGA693, GSE16011, GSE108474, and GSE4412, follow these steps to develop MLTS:

(1) First, we determined the differentially expressed genes (DEGs) between the two clusters of TRPGs in all six cohorts using the "limma" R package, with the filtering criteria for DEGs being adj. p<0.05 and abs(logFC)>1.5. After using univariate COX regression, 11 prognostic TRP-related DEGs specific to gliomas were found in all cohorts.

(2) Next, we applied 10 classic machine learning algorithms, including random forest (RSF), elastic network (Enet), stepwise Cox, Cox boost, Cox partial least squares regression (plsRcox), supervised principal component (SuperPC), gradient enhancement machine (GBM), survival support vector machine (survival-svm), Ridge, and minimum absolute contraction and selection operator (LASSO). Some algorithms can be used as feature selection tools, such as Lasso, stepwise Cox, Cox boost and RSF. Thus, these algorithms were integrated into 96 combinations to fit prognostic models. Ten-fold cross-validation was used to screen the most valuable signatures with the highest C-idex.

(3) The TCGA-Gliomas cohort was used as the training set for 96 algorithms; the other five cohorts served as testing cohorts. We calculated the MLTS for each cohort according to the model obtained from the training cohort.

(4) For each model, the C-index of all testing cohorts was calculated, and the model with the highest average C-index was considered to be the best.

**The prediction of immunotherapy response**

We predicted immunotherapy response to MLTS by analyzing the expression of immune checkpoints, TMB, and TIDE scores. In addition, we used immunotherapy cohorts with comprehensive clinical information to predict immunotherapy responses. Using the “IMvigor210CoreBiologies” R package; the transcriptome data, survival data, and immunotherapy effects of the IMvigor210 cohort were obtained^22^. After eliminating samples without survival time information, 298 samples remained. We downloaded the expression data, clinical information, and immunotherapy outcomes of phs000452, PRJEB23709, and PEJNA482620 cohorts from TIGER website (http://tiger.canceromics.org/). We calculated MLTS for each patient in these cohorts to investigate the impact of MLTS on immunotherapy. Submaps were used to determine associations between high or low MLTS and anti-PD-1 and CTAL4 immune checkpoint therapy^23, 24^.

**The prediction of chemotherapeutic sensitivity**

GDSC data sets were used to predict the drug sensitivity. We used the R software package "prophytic" to predict the relationship between MLTS and the chemotherapeutic drug IC50.

**Cell culture**

The human monocytic leukemia (THP-1) cell line was obtained from the Department of Hematology, Renmin Hospital of Wuhan University. THP-1 cells were cultured in RPMI-1640 medium (Pricella, PM150110) supplemented with 10% heat-inactivated fet bovine serum (FBS, Pricella, 164210) and 1% penicillin–streptomycin (Sigma-Aldrich, V900929). To generate macrophages, THP-1 cells were treated with 100 ng/ml of phorbol 12-myristate 13-acetate (PMA, Sigma-Aldrich, P1585) for 24 h. The mouse microglial (BV2) cell line was obtained from the Department of Neurosurgery at the Zhongnan Hospital, Wuhan University, China. Mouse (GL261) and human (U251) glioma cell lines were obtained from the Department of Neurosurgery, Huzhou Central Hospital. BV2, GL261, and U251 cells were cultured in Dulbecco's Modified Eagle Medium (DMEM; Pricella, PM150210) supplemented with 10% heat-inactivated FBS (Pricella, 164210) and 1% penicillin-streptomycin (Sigma-Aldrich, V900929). All cells were incubated at 37 °C and 5% CO2.

**Transwell assay**

We performed transwell assays in a 24-well compartment with an 8 μm pore insert. First, we seeded GL261 or U251 cells (4 × 10^5^) into the lower compartment with 0.6 mL complete medium, and seeded BV2 or THP-1 cells (2 × 10^5^) into the upper compartment treated with PBS or 2mM Probenecid. The upper inserts were gently placed in a 24-well plate. After 48 h of incubation, the upper inserts were carefully removed and the cells were fixed with 4% glutaraldehyde (Servicebio, G1101) for 10 min. The lower side of the insert was stained with 1% crystal violet (Servicebio, G1014) for 15 min. Excess crystal violet was removed from membrane inserts. Photographs of three random views were captured under a microscope and the number of cells on the lower side of the inserts was counted.

**Intracranial homograft model and imaging**

We purchased eight-week-old female C57BL/6J mice from Silaikejingda (SJA) Laboratory Animal and injected a suspension of 5 × 10^4^ cells (GL261Luc) in 2 μL of PBS intracranially to establish mouse brain tumors (2 mm right lateral, 1 mm frontal to the bregma, and 3 mm deep) as described^25^. One week later, we performed bioluminescence imaging with a PerkinElmer IVIS Lumina 3 system to verify whether the homograft model was successful. The mice were randomly assigned to two groups (n = 5) and treated with PBS or probenecid (200 mg/kg). Seven days later, treatment was administered three times a weekly for three weeks. The mice were monitored regularly and euthanized when they exhibited severe neurological deficiency or weight loss (>20% of their body weight). All animal protocols were performed in accordance with the National Institutes of Health Guide for the Care and Use of Laboratory Animals and approved by the Medical Ethics Committee of Renmin Hospital of Wuhan University.

**Immunofluorescence (IF) staining**

Brain tissue was collected to detect the expression levels of IBA1 (Cell Signaling Technology, 17198) and CD68 (Abcam, ab955). After anesthesia, we perfused the mice with pre-cooled saline through the heart, fixed the brain tissues with 4% paraformaldehyde, and embedded them in paraffin. Then, the embedded tissues were cut into 6-μm sections. Before incubation with the primary antibody, deparaffinization, hydration, antigen repair, membrane rupture, and blocking were performed. After placing the sections incubated with primary antibodies at 4° overnight, we washed them with PBST. Finally, CoraLite488-conjugated Goat Anti-Mouse (Proteintech, SA00013-1) or CoraLite594-conjugated Goat Anti-Rabbit (Proteintech, SA00013-4) antibodies were added and incubated for one h at room temperature. After washing thrice with PBST, the nuclei were stained with DAPI. The number of positively stained cells was calculated and averaged using ImageJ software as previously described^26^. The participants who analyzed the images were blinded to the experimental groups.

**Immunohistochemistry (IHC) staining**

We performed dewaxing of the sections were dewaxed, hydrated with graded alcohol, and subjected to antigen retrieval. Next, the UltraSensitive SP (MXB, KIT-9710) instructions for subsequent operations included blocking endogenous peroxidase, adding non-specific staining blocking reagent, incubating with the primary antibody, adding biotin-labeled IgG polymer, and adding streptavidin-polymer. oxidase. Finally, the slides were mounted after DAB color development and hematoxylin counterstaining.

**Statistical analysis**

All statistical analyses were performed by R software (version 4.0.2). The Wilcoxon rank-sum test was used for the analysis of nonnormally distributed variables between the two groups. The t-test was employed for the statistics of normally distributed variables. Comparisons between three or more groups were conducted using one-way ANOVA and Kruskal-Wallis to test for normally or non-normally distributed variables. Categorical variables were analyzed using the Fisher test and the chi-square test. Correlations were calculated using the spearman and Pearson techniques. Using the Kaplan-Meier method and the Log-Rank test, the survival differences between groups were determined. For the calculation of risk ratios and confidence intervals, we used univariate and multivariate COX. Statistical significance was defined as p < 0.05.

1. Zhang, B.; Wu, Q.; Li, B.; Wang, D.; Wang, L.; Zhou, Y. L., m6A regulator-mediated methylation modification patterns and tumor microenvironment infiltration characterization in gastric cancer. *Mol Cancer* **2020,** *19* (1), 53.

2. Liu, C.-J.; Hu, F.-F.; Xia, M.-X.; Han, L.; Zhang, Q.; Guo, A.-Y., GSCALite: a web server for gene set cancer analysis. *Bioinformatics* **2018,** *34* (21), 3771-3772.

3. Butler, A.; Hoffman, P.; Smibert, P.; Papalexi, E.; Satija, R., Integrating single-cell transcriptomic data across different conditions, technologies, and species. *Nat Biotechnol* **2018,** *36* (5), 411-420.

4. Chen, Z.; Luo, Z.; Zhang, D.; Li, H.; Liu, X.; Zhu, K.; Zhang, H.; Wang, Z.; Zhou, P.; Ren, J.; Zhao, A.; Zuo, Z., TIGER: A Web Portal of Tumor Immunotherapy Gene Expression Resource. *Genomics Proteomics Bioinformatics* **2022**.

5. Yoshihara, K.; Shahmoradgoli, M.; Martínez, E.; Vegesna, R.; Kim, H.; Torres-Garcia, W.; Treviño, V.; Shen, H.; Laird, P. W.; Levine, D. A.; Carter, S. L.; Getz, G.; Stemke-Hale, K.; Mills, G. B.; Verhaak, R. G. W., Inferring tumour purity and stromal and immune cell admixture from expression data. *Nat Commun* **2013,** *4*, 2612.

6. Charoentong, P.; Finotello, F.; Angelova, M.; Mayer, C.; Efremova, M.; Rieder, D.; Hackl, H.; Trajanoski, Z., Pan-cancer Immunogenomic Analyses Reveal Genotype-Immunophenotype Relationships and Predictors of Response to Checkpoint Blockade. *Cell Rep* **2017,** *18* (1), 248-262.

7. Newman, A. M.; Liu, C. L.; Green, M. R.; Gentles, A. J.; Feng, W.; Xu, Y.; Hoang, C. D.; Diehn, M.; Alizadeh, A. A., Robust enumeration of cell subsets from tissue expression profiles. *Nat Methods* **2015,** *12* (5), 453-457.

8. Aran, D.; Hu, Z.; Butte, A. J., xCell: digitally portraying the tissue cellular heterogeneity landscape. *Genome Biol* **2017,** *18* (1), 220.

9. Li, T.; Fan, J.; Wang, B.; Traugh, N.; Chen, Q.; Liu, J. S.; Li, B.; Liu, X. S., TIMER: A Web Server for Comprehensive Analysis of Tumor-Infiltrating Immune Cells. *Cancer Res* **2017,** *77* (21), e108-e110.

10. Finotello, F.; Mayer, C.; Plattner, C.; Laschober, G.; Rieder, D.; Hackl, H.; Krogsdam, A.; Loncova, Z.; Posch, W.; Wilflingseder, D.; Sopper, S.; Ijsselsteijn, M.; Brouwer, T. P.; Johnson, D.; Xu, Y.; Wang, Y.; Sanders, M. E.; Estrada, M. V.; Ericsson-Gonzalez, P.; Charoentong, P.; Balko, J.; de Miranda, N. F. d. C. C.; Trajanoski, Z., Molecular and pharmacological modulators of the tumor immune contexture revealed by deconvolution of RNA-seq data. *Genome Med* **2019,** *11* (1), 34.

11. Becht, E.; Giraldo, N. A.; Lacroix, L.; Buttard, B.; Elarouci, N.; Petitprez, F.; Selves, J.; Laurent-Puig, P.; Sautès-Fridman, C.; Fridman, W. H.; de Reyniès, A., Estimating the population abundance of tissue-infiltrating immune and stromal cell populations using gene expression. *Genome Biol* **2016,** *17* (1), 218.

12. Xiao, Y.; Ma, D.; Zhao, S.; Suo, C.; Shi, J.; Xue, M.-Z.; Ruan, M.; Wang, H.; Zhao, J.; Li, Q.; Wang, P.; Shi, L.; Yang, W.-T.; Huang, W.; Hu, X.; Yu, K.-D.; Huang, S.; Bertucci, F.; Jiang, Y.-Z.; Shao, Z.-M., Multi-Omics Profiling Reveals Distinct Microenvironment Characterization and Suggests Immune Escape Mechanisms of Triple-Negative Breast Cancer. *Clin Cancer Res* **2019,** *25* (16), 5002-5014.

13. Jiang, P.; Gu, S.; Pan, D.; Fu, J.; Sahu, A.; Hu, X.; Li, Z.; Traugh, N.; Bu, X.; Li, B.; Liu, J.; Freeman, G. J.; Brown, M. A.; Wucherpfennig, K. W.; Liu, X. S., Signatures of T cell dysfunction and exclusion predict cancer immunotherapy response. *Nat Med* **2018,** *24* (10), 1550-1558.

14. Zeng, D.; Ye, Z.; Shen, R.; Yu, G.; Wu, J.; Xiong, Y.; Zhou, R.; Qiu, W.; Huang, N.; Sun, L.; Li, X.; Bin, J.; Liao, Y.; Shi, M.; Liao, W., IOBR: Multi-Omics Immuno-Oncology Biological Research to Decode Tumor Microenvironment and Signatures. *Front Immunol* **2021,** *12*, 687975.

15. Xu, L.; Deng, C.; Pang, B.; Zhang, X.; Liu, W.; Liao, G.; Yuan, H.; Cheng, P.; Li, F.; Long, Z.; Yan, M.; Zhao, T.; Xiao, Y.; Li, X., TIP: A Web Server for Resolving Tumor Immunophenotype Profiling. *Cancer Res* **2018,** *78* (23), 6575-6580.

16. Akbani, R.; Ng, P. K. S.; Werner, H. M. J.; Shahmoradgoli, M.; Zhang, F.; Ju, Z.; Liu, W.; Yang, J.-Y.; Yoshihara, K.; Li, J.; Ling, S.; Seviour, E. G.; Ram, P. T.; Minna, J. D.; Diao, L.; Tong, P.; Heymach, J. V.; Hill, S. M.; Dondelinger, F.; Städler, N.; Byers, L. A.; Meric-Bernstam, F.; Weinstein, J. N.; Broom, B. M.; Verhaak, R. G. W.; Liang, H.; Mukherjee, S.; Lu, Y.; Mills, G. B., A pan-cancer proteomic perspective on The Cancer Genome Atlas. *Nat Commun* **2014,** *5*, 3887.

17. Ye, Y.; Xiang, Y.; Ozguc, F. M.; Kim, Y.; Liu, C.-J.; Park, P. K.; Hu, Q.; Diao, L.; Lou, Y.; Lin, C.; Guo, A.-Y.; Zhou, B.; Wang, L.; Chen, Z.; Takahashi, J. S.; Mills, G. B.; Yoo, S.-H.; Han, L., The Genomic Landscape and Pharmacogenomic Interactions of Clock Genes in Cancer Chronotherapy. *Cell Syst* **2018,** *6* (3).

18. Hänzelmann, S.; Castelo, R.; Guinney, J., GSVA: gene set variation analysis for microarray and RNA-seq data. *BMC Bioinformatics* **2013,** *14*, 7.

19. Wang, L.; Liu, Z.; Liang, R.; Wang, W.; Zhu, R.; Li, J.; Xing, Z.; Weng, S.; Han, X.; Sun, Y.-L., Comprehensive machine-learning survival framework develops a consensus model in large-scale multicenter cohorts for pancreatic cancer. *Elife* **2022,** *11*.

20. Liu, Z.; Liu, L.; Weng, S.; Guo, C.; Dang, Q.; Xu, H.; Wang, L.; Lu, T.; Zhang, Y.; Sun, Z.; Han, X., Machine learning-based integration develops an immune-derived lncRNA signature for improving outcomes in colorectal cancer. *Nat Commun* **2022,** *13* (1), 816.

21. Liu, Z.; Guo, C.; Dang, Q.; Wang, L.; Liu, L.; Weng, S.; Xu, H.; Lu, T.; Sun, Z.; Han, X., Integrative analysis from multi-center studies identities a consensus machine learning-derived lncRNA signature for stage II/III colorectal cancer. *EBioMedicine* **2022,** *75*, 103750.

22. Mariathasan, S.; Turley, S. J.; Nickles, D.; Castiglioni, A.; Yuen, K.; Wang, Y.; Kadel, E. E.; Koeppen, H.; Astarita, J. L.; Cubas, R.; Jhunjhunwala, S.; Banchereau, R.; Yang, Y.; Guan, Y.; Chalouni, C.; Ziai, J.; Şenbabaoğlu, Y.; Santoro, S.; Sheinson, D.; Hung, J.; Giltnane, J. M.; Pierce, A. A.; Mesh, K.; Lianoglou, S.; Riegler, J.; Carano, R. A. D.; Eriksson, P.; Höglund, M.; Somarriba, L.; Halligan, D. L.; van der Heijden, M. S.; Loriot, Y.; Rosenberg, J. E.; Fong, L.; Mellman, I.; Chen, D. S.; Green, M.; Derleth, C.; Fine, G. D.; Hegde, P. S.; Bourgon, R.; Powles, T., TGFβ attenuates tumour response to PD-L1 blockade by contributing to exclusion of T cells. *Nature* **2018,** *554* (7693), 544-548.

23. Hoshida, Y.; Brunet, J.-P.; Tamayo, P.; Golub, T. R.; Mesirov, J. P., Subclass mapping: identifying common subtypes in independent disease data sets. *PLoS One* **2007,** *2* (11), e1195.

24. Lu, X.; Jiang, L.; Zhang, L.; Zhu, Y.; Hu, W.; Wang, J.; Ruan, X.; Xu, Z.; Meng, X.; Gao, J.; Su, X.; Yan, F., Immune Signature-Based Subtypes of Cervical Squamous Cell Carcinoma Tightly Associated with Human Papillomavirus Type 16 Expression, Molecular Features, and Clinical Outcome. *Neoplasia* **2019,** *21* (6), 591-601.

25. Berger, G.; Knelson, E. H.; Jimenez-Macias, J. L.; Nowicki, M. O.; Han, S.; Panagioti, E.; Lizotte, P. H.; Adu-Berchie, K.; Stafford, A.; Dimitrakakis, N.; Zhou, L.; Chiocca, E. A.; Mooney, D. J.; Barbie, D. A.; Lawler, S. E., STING activation promotes robust immune response and NK cell-mediated tumor regression in glioblastoma models. *Proc Natl Acad Sci U S A* **2022,** *119* (28), e2111003119.

26. Zhang, Y.; Ye, P.; Zhu, H.; Gu, L.; Li, Y.; Feng, S.; Zeng, Z.; Chen, Q.; Zhou, B.; Xiong, X., Neutral polysaccharide from Gastrodia elata alleviates cerebral ischemia-reperfusion injury by inhibiting ferroptosis-mediated neuroinflammation via the NRF2/HO-1 signaling pathway. *CNS Neurosci Ther* **2023**.

**Supplementary figures**

**
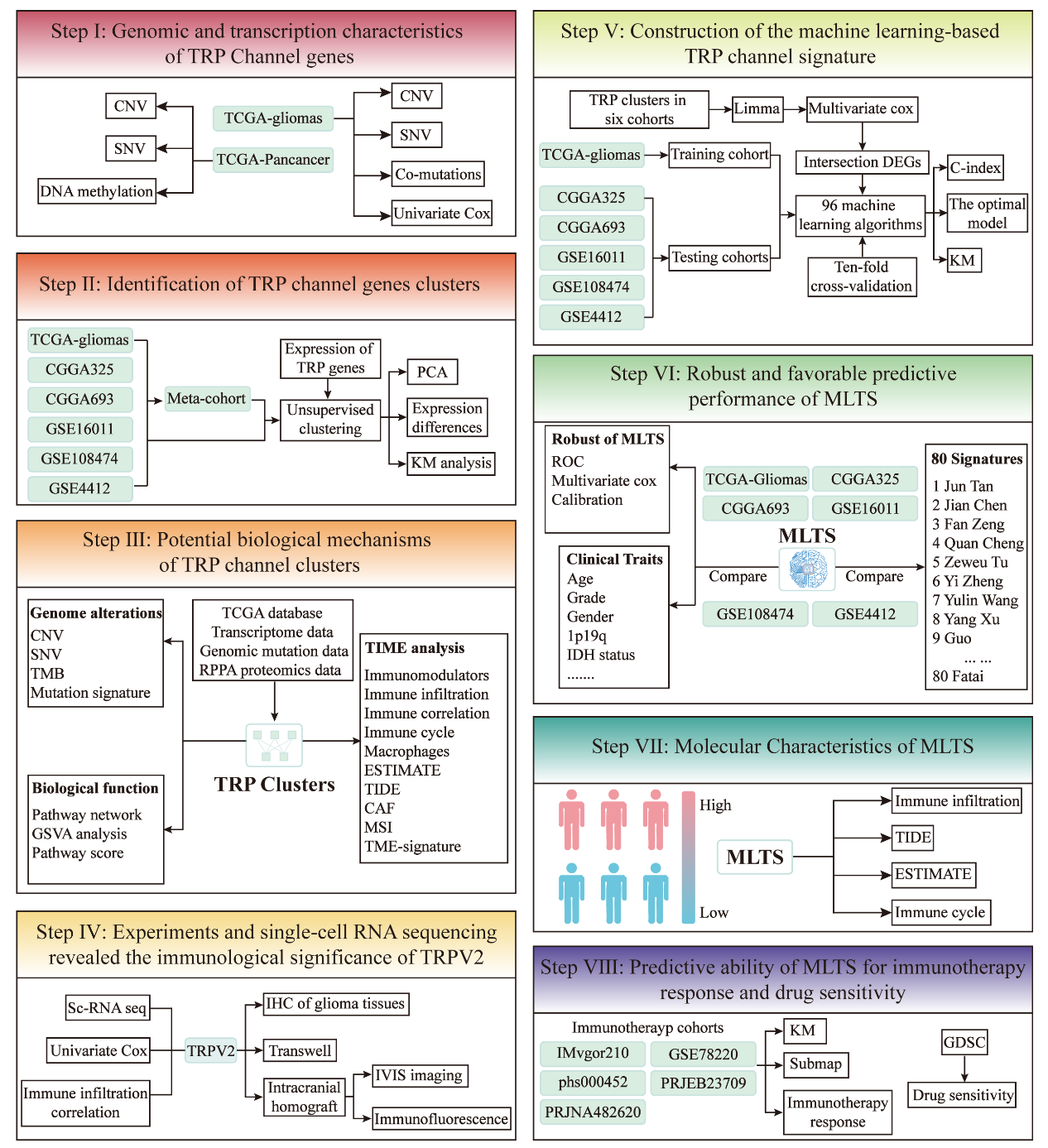
**

**Figure S1. The workflow of this study.**


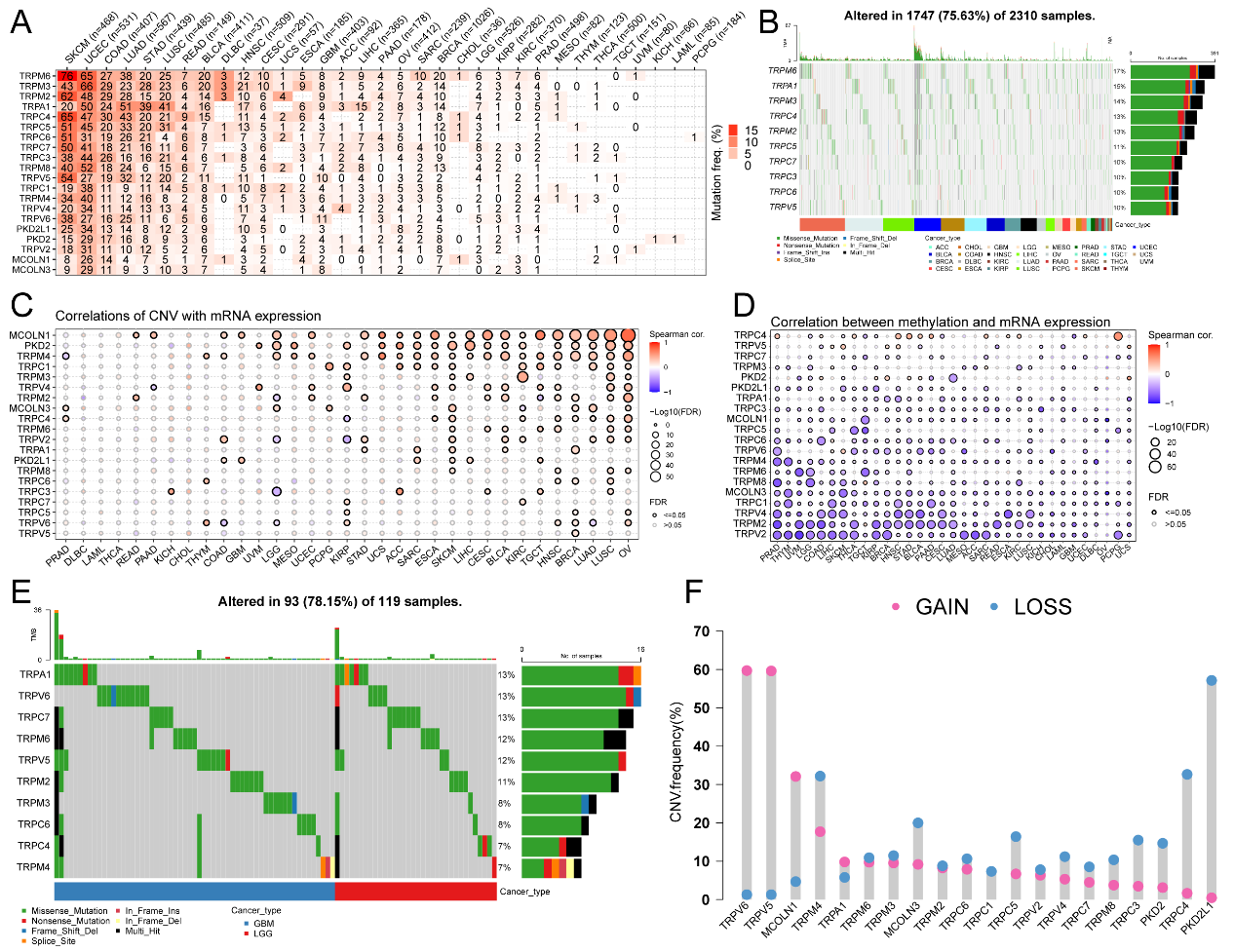


**Figure S2. Altered epigenetic landscape of TRPGs in pan-cancer and gliomas.**

(A) The mutation frequency heatmap revealed the mutation rate of TRPGs in pan-cancer. The blanks indicates that the gene has no mutation. (B) The waterfall plot showed the mutation frequency and types of TRPGs in 32 cancers from the TCGA cohort. (C) The heat map displayed the correlation between mRNA levels of TRPGs and copy number variation. (D) The heat map displayed the correlation between mRNA levels of TRPGs and DNA methylation. Red and blue indicate positive and negative correlations, respectively. The deeper the color, the greater the correlation. Bigger bubble indicates a lower the p-value. (E) The waterfall plot showed the mutation frequency and types of TRPGs in glioma patients. (F) The CNV frequency of TRPGs in glioma patients from the TCGA cohort. Pink and blue dots indicate CNV frequency gained and lost, respectively.


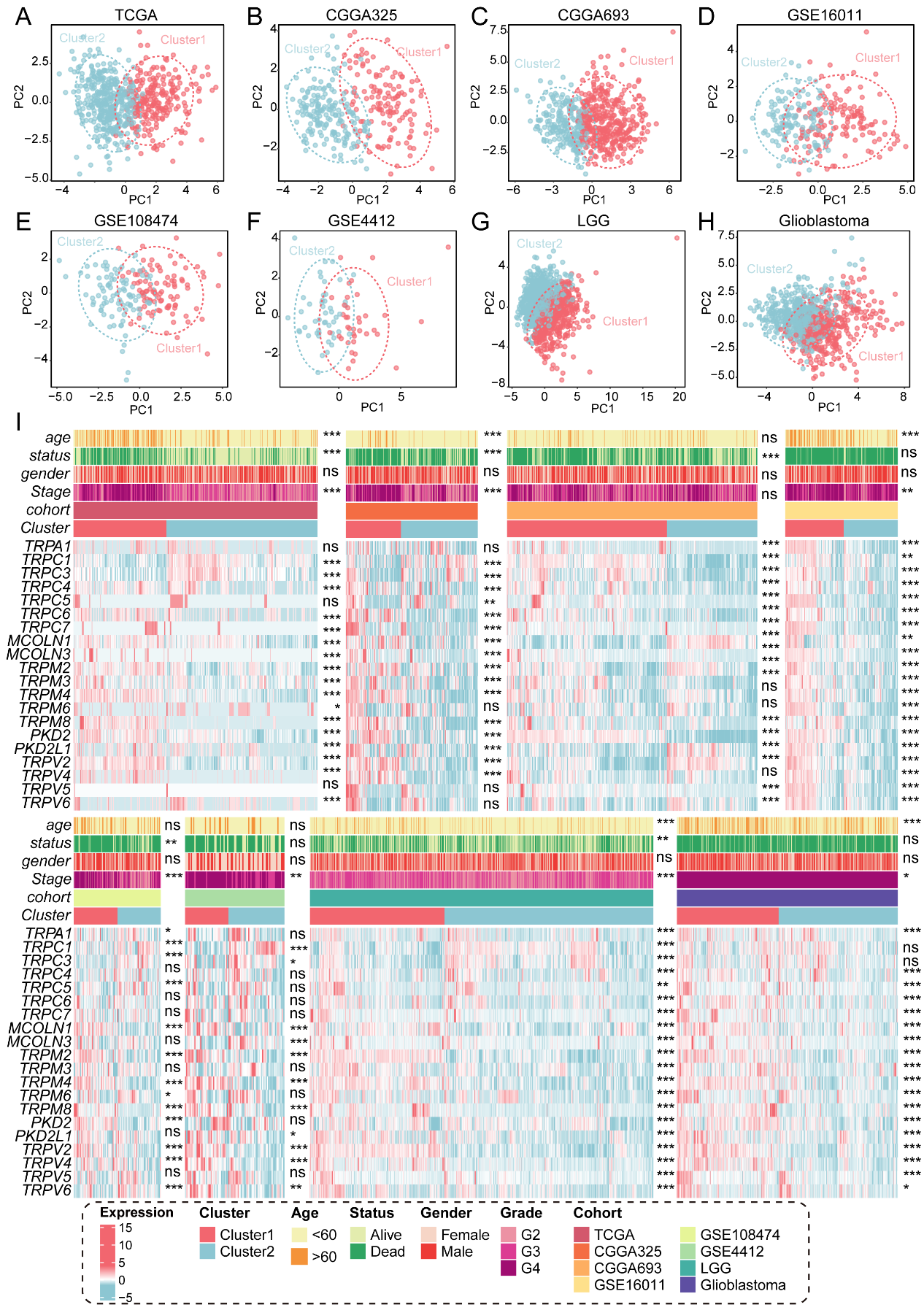


**Figure S3. Track of the unsupervised K-means-based consensus-clustering.**

(A-H) PCA for the transcriptome profiles of TRP channel clusters in the TCGA (A), CGGA325 (B), CGGA693 (C), GSE16011 (D), GSE108474 (E), GSE4412 (F), LGG (G), and Glioblastoma (H) cohorts. (I) This heatmap demonstrates the relationships between the two TRP channel clusters, clinicopathologic characteristics, and the expression variations of the TRPGs in eight cohorts. The top portion represented Fisher's precise test. The lower portion indicated the Wilcoxon rank-sum test. ****p* < 0.001, ***p* < 0.01, **p* < 0.05, and "ns" stood for no statistical significance.


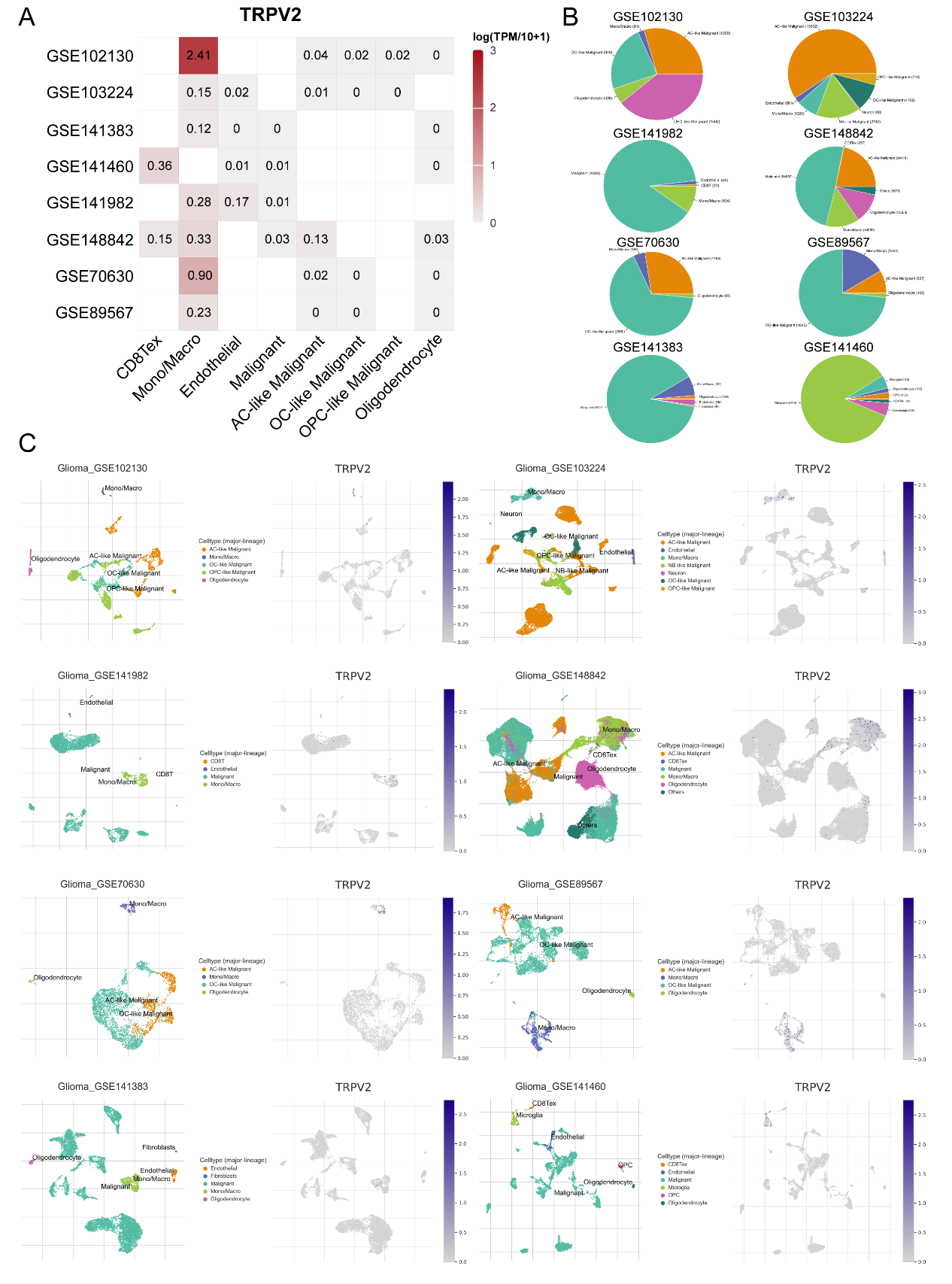


**Figure S4. Expression levels of TRPV2 in different cell types in single-cell RNA sequencing datasets.**

(A) The heatmap showed TRPV2 expression in different glioma single-cell RNA sequencing datasets. (B) The pie plots showed the cell type percentages in GSE102130, GSE103224, GSE141383, GSE141460, GSE141982, GSE148842, GSE70630, and GSE89567. (C) the UMAP plots illustrated the expression of CDCA2 in different cell types based on the GSE102130, GSE103224, GSE141383, GSE141460, GSE141982, GSE148842, GSE70630, and GSE89567.


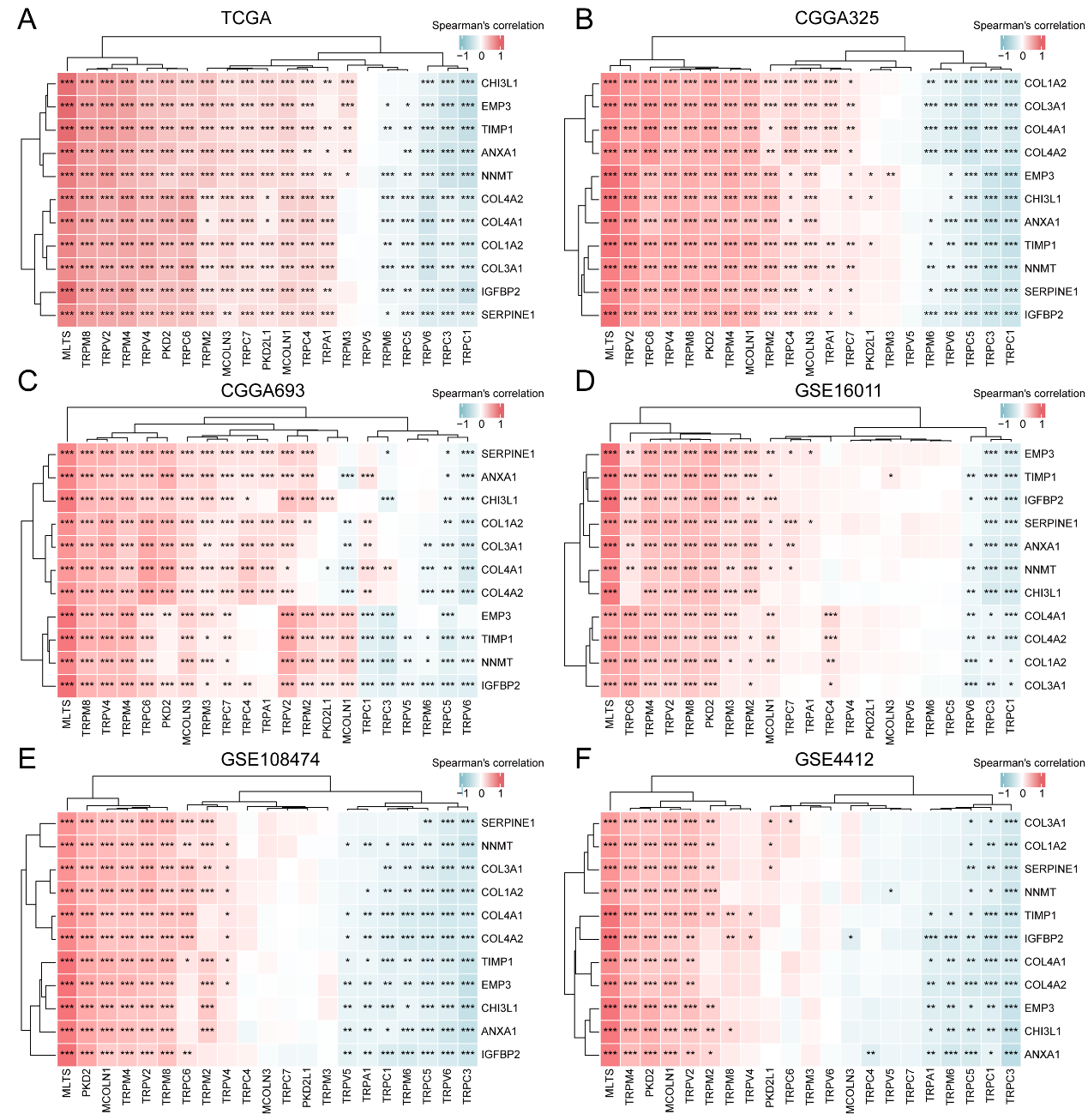


**Figure S5. The correlation between TRPGs, MLTS and the TRP-related DEGs.**

(A-F) The heatmap exhibited the correlation between mRNA expression levels of TRPGs, MLTS and the 11 prognostic TRP-related genes specific to gliomas. Asterisks denoted p-value (**p* < 0.05; ***p* < 0.01, ****p* < 0.001). Blank cells represented no statistical significance of the correlation.
